# Synthesis of benzo[*d*]imidazo[2,1-*b*]benzoselenoazoles: Cs_2_CO_3_-mediated cyclization of 1-(2-bromoaryl)benzimidazoles with selenium

**DOI:** 10.3762/bjoc.15.199

**Published:** 2019-08-26

**Authors:** Mio Matsumura, Yuki Kitamura, Arisa Yamauchi, Yoshitaka Kanazawa, Yuki Murata, Tadashi Hyodo, Kentaro Yamaguchi, Shuji Yasuike

**Affiliations:** 1 School of Pharmaceutical Sciences, Aichi Gakuin University, 1-100 Kusumoto-cho, Chikusa-ku, Nagoya 464-8650, Japan; 2 Pharmaceutical Sciences at Kagawa Campus, Tokushima Bunri University, 1314-1 Shido, Sanuki, Kagawa 769-2193, Japan

**Keywords:** benzimidazole, cesium carbonate, cyclization, selenium, selenoazole

## Abstract

The synthesis of benzimidazo[2,1-*b*]benzoselenoazoles is described. The novel ring-closure reaction of 1-(2-bromoaryl)benzimidazoles with Se powder is promoted by Cs_2_CO_3_ (2 equiv) in DMF at 150 °C. Moreover, the obtained tetracyclic heterocycles are all novel compounds. Single-crystal X-ray analysis of the parent benzimidazo[2,1-*b*]benzoselenoazole revealed that the tetracyclic ring is almost planar. Absorption spectroscopy data of the benzimidazo[2,1-*b*]benzoselenoazoles showed the λ_max_ was dependent on the number of rings.

## Introduction

**Scheme 1:**
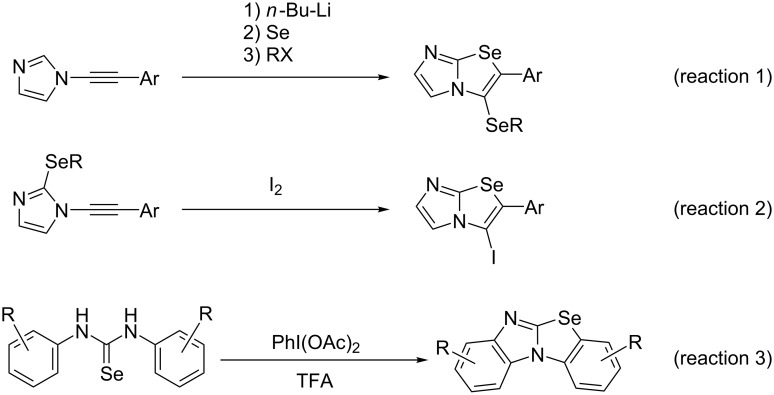
Previously reported synthetic methods for the preparation of imidazo[2,1-*b*]selenoazoles.

Selenium-containing heterocyclic ring systems have attracted attention not only because of their chemical properties and reactivities, but also for their wide biological activities [1–4]. For example, imidazo[2,1-*b*]selenoazoles, in which imidazole and selenophene are condensed, have been synthesized, and it was described in a patent that imidazo[2,1-*b*]benzoselenazole-3-acetamide derivatives have anticonvulsant activity [5]. Three ring closure reactions for the synthesis of imidazo[2,1-*b*]selenoazole have been reported so far (Scheme 1). Zeni et al. reported the synthesis of imidazoselenoazole using a three-step one-pot reaction of *N*-alkynylimidazoles with selenium involving the electrophilic intramolecular cyclization of acetylenic compounds (Scheme 1, reaction 1) [6]. Zeni et al. also developed a ring closure reaction using *N*-alkynyl-2-alkylselanylimidazoles and I_2_ (Scheme 1, reaction 2) [7]. Moreover, Punniyamurthy et al. reported the synthesis of benzo[*d*]imidazo[2,1-*b*]benzoselenoazoles using an oxidative cyclization by reacting 1,3-diarylselenourea with (diacetoxyiodo)benzene (Scheme 1, reaction 3) [8]. However, to the best of our knowledge, the synthesis of imidazoselenoazoles has been limited to highly substituted derivatives and the basic physical properties of the parent skeleton have not been clarified.

Transition metal-catalyzed reactions are one of the most popular methods to form Ar(aryl)–Se bonds [9–13]. Various metals, such as Pd, Ni, Fe, and Cu have been used to catalyze the reactions of a Se source with aryl donors. Among these, Cu-catalyzed tandem cyclization via a one-step Ullmann-type Se-arylation and C_sp2_–H selenation are efficient methods for constructing tetracyclic aromatic heterocycles containing selenium. For example, the reaction of 2-(2-iodophenyl)indoles with selenium powder in the presence of CuO as catalyst resulted in benzoselenopheno[3,2-*b*]indole derivatives [14]. The reaction of 2-(2-haloaryl)imidazo[1,2-*a*]pyridines with selenium using a CuI catalyst for the synthesis of benzo[*b*]selenophene-fused imidazo[1,2-*a*]pyridines occurred smoothly [15–16]. Performing these types of reactions without the addition of a transition metal catalyst is more challenging, but would alleviate the environmental burden of removing and disposing of the metal catalyst. We present in this paper the synthesis of benzo[*d*]imidazo[2,1-*b*]benzoselenoazoles under transition metal-free conditions by the Cs_2_CO_3_-mediated cyclization of 1-(2-bromoaryl)benzimidazoles with selenium.

## Results and Discussion

We initially focused our attention on determining the optimal conditions for the cyclization of a chalcogen with 1-(2-bromophenyl)benzimidazole (**1a**). Table 1 shows the results from the screening of additives, solvents, and chalcogens. Since most of these types of reactions require a transition metal catalyst such as a copper reagent [14–16], the reaction between **1a** and Se powder was initially carried out using CuI (10 mol %) and Cs_2_CO_3_ (2 equiv) in DMF at 150 °C under an argon atmosphere to obtain the parent tetracyclic benzimidazo[2,1-*b*]benzoselenoazole (**2a**) in 64% yield (Table 1, entry 1). Surprisingly, the yield of **2a** improved significantly when the copper catalyst was not present (Table 1, entry 2). Several bases were screened for the reaction of **1a** with Se powder (Table 1, entries 2–8). The use of Cs_2_CO_3_ resulted in the highest yield of **2a** (99%, Table 1, entry 2). Decreasing the loading of Cs_2_CO_3_ from 2 to 1 equivalent significantly reduced the yield of **2a** (Table 1, entry 9), and the reaction did not proceed in the absence of a base (Table 1, entry 10). After optimizing the choice and quantity of base, a solvent screening showed that the reaction proceeded efficiently in DMF (99%), and DMSO (73%), whereas the use of NMP, toluene, dioxane, and 1,2-DCE resulted in inefficient reactions (Table 1, entries 2 and 11–15). Under aerobic conditions, a significant decrease in the yield of product **2a** was observed (Table 1, entry 16). We also attempted the cyclization of 1-(2-bromophenyl)benzimidazoles **1a** using other chalcogen powders. However, the reaction of **1a** with sulfur or tellurium powder did not proceed, and the starting material **1a** was recovered (Table 1, entries 17 and 18). The best result was obtained when **1a** and Se powder were treated with Cs_2_CO_3_ in DMF under an argon atmosphere at 150 °C.

**Table 1:** Cyclization of 1-(2-bromophenyl)benzimidazoles with chalcogen elements.^a^

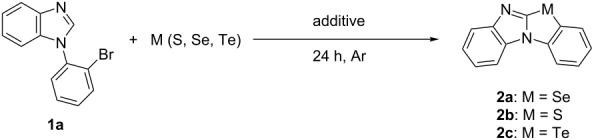					

Entry	M	Additive	Solvent	Temp. (°C)	Yield (%)^b^

1	Se	CuI (10 mol %), Cs_2_CO_3_ (2 equiv)	DMF	150	**2a**: 64
2	Se	Cs_2_CO_3_ (2 equiv)	DMF	150	**2a**: 99 (93)^c^
3	Se	Na_2_CO_3_ (2 equiv)	DMF	150	**2a**: 12
4	Se	CsOH (2 equiv)	DMF	150	**2a**: 28
5	Se	K_3_PO_4_ (2 equiv)	DMF	150	**2a**: 67
6	Se	KOAc (2 equiv)	DMF	150	**2a**: 70
7	Se	*t*-BuOLi (2 equiv)	DMF	150	**2a**: 38
8	Se	*t*-BuOK (2 equiv)	DMF	150	**2a**: 34
9	Se	Cs_2_CO_3_ (1 equiv)	DMF	150	**2a**: 70
10	Se	–	DMF	150	**2a**: 0
11	Se	Cs_2_CO_3_ (2 equiv)	DMSO	150	**2a**: 73
12	Se	Cs_2_CO_3_ (2 equiv)	NMP	150	**2a**: 25
13	Se	Cs_2_CO_3_ (2 equiv)	toluene	110	**2a**: 0
14	Se	Cs_2_CO_3_ (2 equiv)	dioxane	80	**2a**: 0
15	Se	Cs_2_CO_3_ (2 equiv)	1,2-DCE	80	**2a**: 0
16^d^	Se	Cs_2_CO_3_ (2 equiv)	DMF	150	**2a**: 23
17	S	Cs_2_CO_3_ (2 equiv)	DMF	150	**2b**: 0
18	Te	Cs_2_CO_3_ (2 equiv)	DMF	150	**2c**: 0

^a^Reaction conditions: **1a** (0.5 mmol), chalcogen element (1 mmol). ^b^GC yield using dibenzyl as internal standard. ^c^Isolated yiels. ^d^Under air.

**Figure 1:**
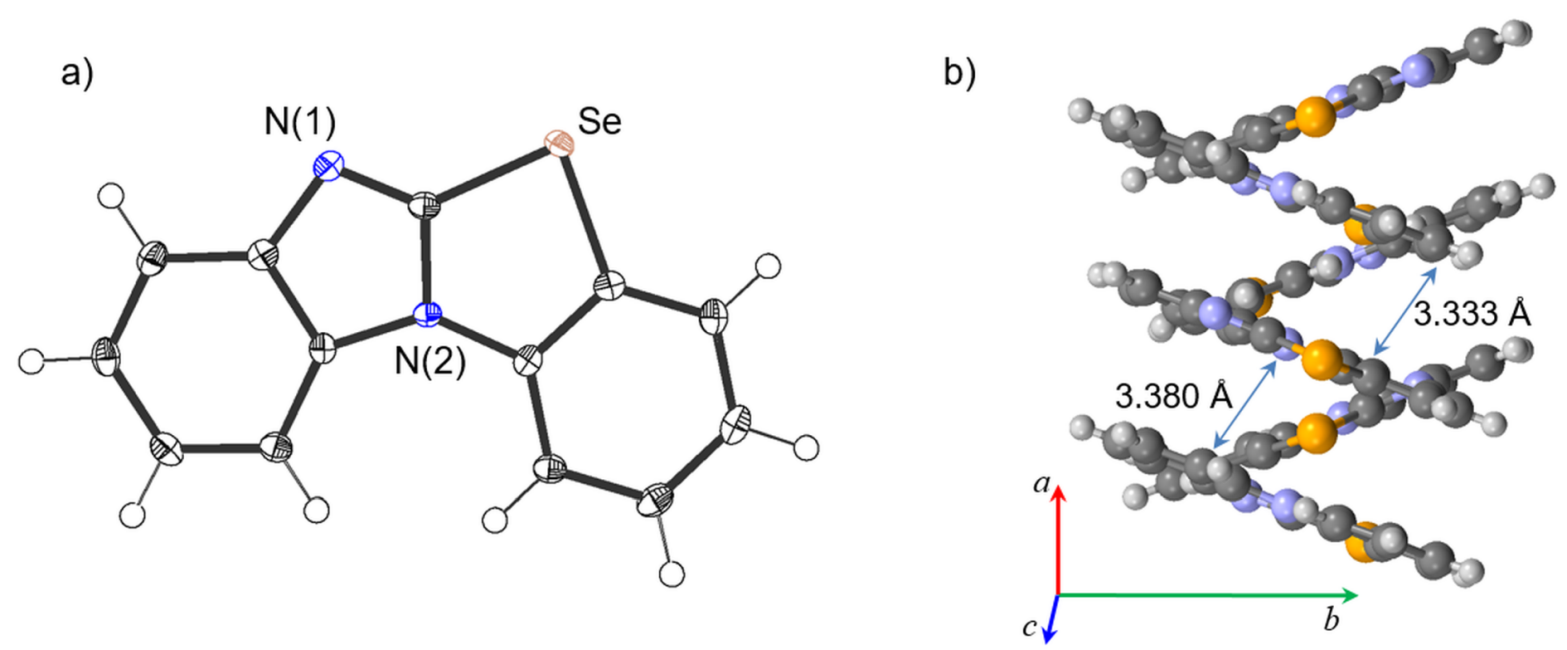
(a) Ortep drawing of **2a** (50% probability, only one of two independent molecules is shown) and (b) packing structure. The component based solvent was omitted for clarity.

The benzimidazo[2,1-*b*]benzoselenoazole product **2a** was fully characterized by ^1^H and ^13^C NMR spectroscopy and HRMS, and further confirmed by single-crystal X-ray diffraction (XRD) analysis. The ORTEP drawing and packing structure of **2a** obtained from the single crystal XRD analysis are illustrated in Figure 1. The crystal structure contained two independent molecules, and the benzimidazole and the fused benzoselenophene rings are virtually coplanar (mean deviation 0.0169 and 0.0359 Å, respectively) to each other. The molecules show head-to-tail (antiparallel) stacking with π···π interactions, with distances between the nearest neighbor atoms on adjacent molecules being 3.333 (C8–C24) and 3.380 Å (C18–C13) (Figure 1b). Moreover, there were intermolecular interactions between Se(1) and N(3) atoms, and the Se(1)‒N(3) distance was 3.133 Å, which was 86% of the sum of the van der Waals radii (3.54 Å) of both elements (see Supporting Information File 1, Figure S1).

**Figure 2:**
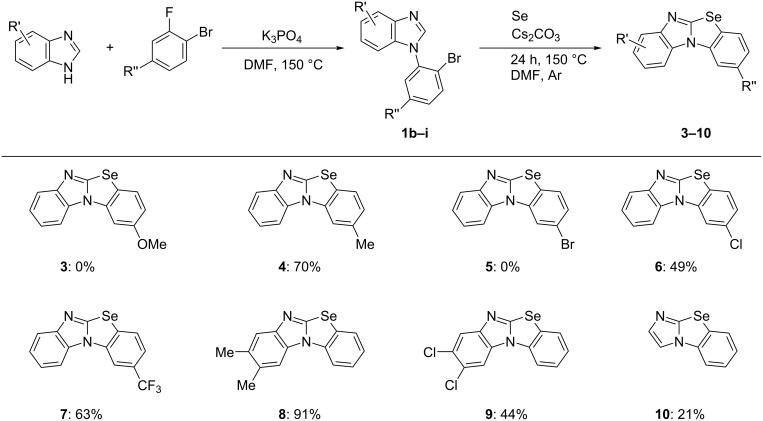
Cs_2_CO_3_-mediated cyclization of 1-(2-bromoaryl)imidazoles with Se. Reaction conditions: **1** (0.5 mmol), Se (1 mmol), Cs_2_CO_3_ (1 mmol), DMF (1 mL), 150 °C, 24 h. Isolated yield.

To demonstrate the efficiency and generality of this cyclization, the reactions of various 1-(2-bromoaryl)benzimidazoles **1b–i** (0.5 mmol) and Se powder (1 mmol) were investigated in DMF in the presence of Cs_2_CO_3_ (1 mmol) at 150 °C. The key 1-(2-bromoaryl)benzimidazole starting materials **1** could be easily prepared according to a previously reported general method [17]. The *N*-arylation of benzo[*d*]imidazoles with 1-bromo-2-fluorobenzene derivatives in the presence of K_3_PO_4_ (5 equiv) at 150 °C gave **1a–i** in 45–99% yields. All synthetic details including the preparation method for 1-(2-bromoaryl)benzimidazoles **1a–i** are given in Supporting Information File 1. The results of the cyclization are summarized in Figure 2. Products **4**, **7**, and **8**, substituted with methyl and trifluoromethyl groups, were obtained in good yield. In contrast, compounds **6** and **9**, substituted with chloro groups, had low solubility in solvents, resulting in only moderate yield. Moreover, a complex mixture was obtained from substrates having a methoxy or bromo group, and the corresponding products **3** and **5** could not be obtained. This may have been caused by the substituents being damaged by the base. The reaction of 1-(2-bromophenyl)imidazole (**1i**) with Se powder gave the corresponding tricyclic product **10** in low yield. Since the reaction conditions for the synthesis of the starting material **1** and the cyclization of **1** with Se powder are similar, we carried out a three-component reaction of benzimidazole, 1-bromo-2-fluorobenzene, and Se powder under the optimized conditions, i.e., in the presence of Cs_2_CO_3_ (2 equiv) in DMF under an argon atmosphere at 150 °C. This reaction gave product **2a** in only 35% yield, suggesting that the stepwise reaction via 1-(2-bromoaryl)benzimidazole **1** is superior.

**Figure 3:**
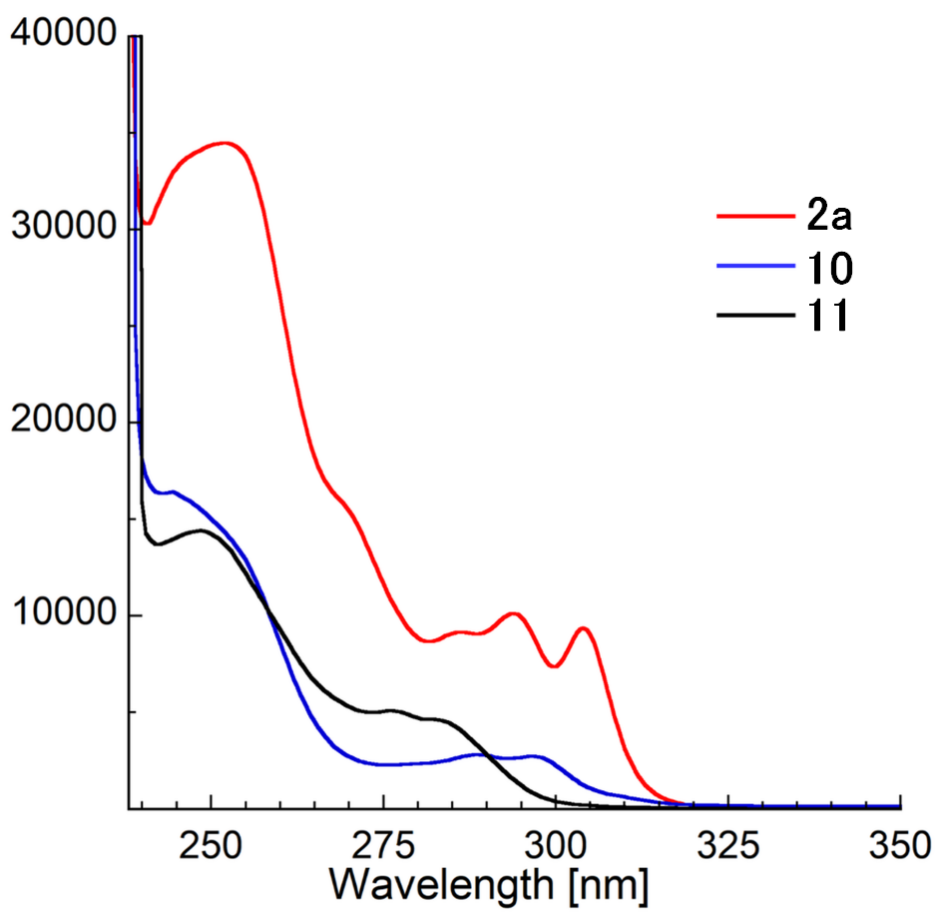
Absorption spectra of selected compounds (**2a**, **10** and **11**) in CHCl_3_.

Next, the photophysical properties of the synthesized compounds were evaluated, and the corresponding data are shown in Table 2 and Figure 3. 1-Phenylbenzimidazole (**11**), which does not contain a selenium atom, has an absorption maximum at 283 nm. In contrast, the maximum absorption wavelength (λ_max_) of parent compound **2a** was found to be 304 nm, which is red-shifted by 21 nm compared with that of **11**. The tricyclic compound **10** has a shorter λ_max_ (297 nm). These results indicate the λ_max_ is dependent on the number of rings. In 2-substituted derivatives (**4**–**9**), the maximum absorptions were very similar to each other (Table 2 and Figure S2 in Supporting Information File 1).

**Table 2:** Absorption spectroscopy data^a^.

Compd.	λ_max_ (ε)	Compd.	λ_max_ (ε)

**2a**	304 (9400)	**8**	301 (9100)
**4**	308 (12900)	**9**	306 (31900)
**6**	313 (9900)	**10**	297 (2700)
**7**	309 (14700)	**11**^b^	283 (4600)

^a^Measured in CHCl_3_. ^b^1-Phenylbenzimidazole.

**Scheme 2:**
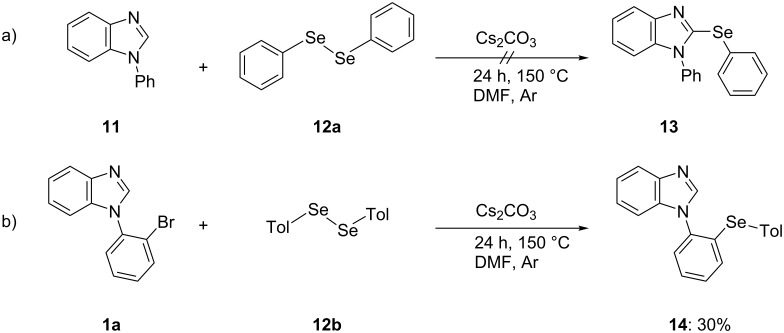
Control reactions.

Cs_2_CO_3_-mediated C(Het)–S bond formations of a heteroazole such as imidazo[1,2-*a*]pyridine, oxadiazole, and benzimidazole with diaryl disulfides without a transition metal catalyst have previously been developed [18–19]. The key step in these reactions is probably deprotonation of the heterocyclic rings with a base. Moreover, nucleophilic aromatic substitution (S_N_Ar) reactions between an aryl halide and a selenium reagent such as aryl selenide anion or diaryl diselenide for C(Ar)–Se bond formation using a base have been reported [20–22]. However, the reaction mechanisms for these syntheses have not been reported. Therefore, we carried out several control experiments to clarify the reaction mechanism (Scheme 2). However, the reaction of 1-phenylbenzimidazole (**11**) without bromine at the phenyl group with diphenyl diselenide (**12a**) did not afford the corresponding 1-phenyl-2-(phenylselanyl)benzimidazole (**13**), and the reaction between 1-(2-bromophenyl)benzimidazole (**1a**) and di-*p*-tolyl diselenide (**12b**) gave 1-[2-(*p*-tolylselanyl)phenyl]benzimidazole (**14**) in only 30% yield.

**Scheme 3:**
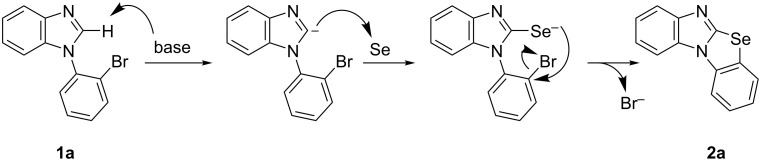
Proposed mechanism.

At present, the mechanism of this cyclization is unclear. We assume the reaction mechanism is as depicted in Scheme 3. The base deprotonates the imidazole ring giving an anion at the 2-position, which reacts with selenium by nucleophilic attack, resulting in C(Het)–Se bond formation. Next, the ring closure proceeds via the S_N_Ar reaction by attack of the selenide anion on the phenyl group having bromine to generate the tetracyclic target molecule.

## Conclusion

Benzo[*d*]imidazo[2,1-*b*]benzoselenoazoles were prepared via Cs_2_CO_3_-mediated tandem cyclization followed by reaction of 1-(2-bromoaryl)benzimidazoles with Se powder without a transition metal catalyst. The molecular structure of parent tetracyclic compound **2a** features a nearly coplanar ring. Absorption spectroscopy data revealed the λ_max_ was dependent on the number of rings. Detailed mechanistic studies of this cyclization and the applications of this reaction to other heterocycles are currently underway in our laboratory.

## Experimental

**General procedure for the synthesis of benzoimidazo[2,1-*b*]benzoselenoazoles:** 1-(2-Bromoaryl)benzimidazoles **1** (0.5 mmol), selenium powder (79 mg, 1.0 mmol, 2 equiv), and cesium carbonate (326 mg, 1.0 mmol, 2 equiv) were dissolved in DMF (1 mL) under Ar atmosphere. The mixture was stirred at 150 °C for 24 h. The reaction mixture was diluted with CH_2_Cl_2_ (15 mL) and water (15 mL). The phases were separated, and the aqueous layer was extracted with CH_2_Cl_2_ (2 × 10 mL). The combined organic layers were washed with water (2 × 20 mL), dried with MgSO_4_, and concentrated under reduced pressure. The residue was purified by silica gel chromatography (*n-*hexane/AcOEt).

## Supporting Information

File 1 Experimental details and analytical data, copies of absorption and NMR spectra.

File 2 X-ray crystal structure of **2a**.
