# Supplementary material for: Synthesis of benzo[d]imidazo[2,1-b]benzoselenoazoles: Cs2CO3-mediated cyclization of 1-(2-bromoaryl)benzimidazoles with selenium
Source: Beilstein J Org Chem. 2019 Aug 26;15:2029–35. doi: 10.3762/bjoc.15.199 (PMC6720659; doi:10.3762/bjoc.15.199)

BEILSTEIN JOURNAL OF ORGANIC CHEMISTRY

Supporting Information

for

Synthesis of benzo[*d*]imidazo[2,1-*b*]benzoselenoazoles: Cs_2CO_3-mediated cyclization of 1-(2-bromoaryl)benzimidazoles with selenium

Mio Matsumura, Yuki Kitamura, Arisa Yamauchi, Yoshitaka Kanazawa, Yuki Murata, Tadashi Hyodo, Kentaro Yamaguchi and Shuji Yasuike

Beilstein J. Org. Chem. **2019,** *15,* 2029–2035. doi:10.3762/bjoc.15.199

Experimental details and analytical data, copies of absorption and NMR spectra

License and Terms: This is a supporting information file under the terms of the Creative Commons Attribution License (http://creativecommons.org/licenses/by/4.0). Please note that the reuse, redistribution and reproduction in particular requires that the authors and source are credited.
The license is subject to the *Beilstein Journal of Organic Chemistry* terms and conditions: (https://www.beilstein-journals.org/bjoc)

Contents

1. General information S2

2. Experimental details and characterization data S2

3. X-ray crystal structure determinations of **2a** S8

4. Absorption spectra of benzoimidazo[2,1-*b*]benzoselenoazoles S9

5. References S9

6. NMR spectra of new compounds S10

1. General information

Melting points were measured on a Yanagimoto micro melting point hot-stage apparatus (MP-S3) and reported as uncorrected values. ^{1}H NMR (400 MHz, TMS: δ: 0.00 ppm as an internal standard), ^{13}C NMR (100 MHz, $CDCl_3$: δ: 77.00 or DMSO-d_6: δ: 39.52 ppm as an internal standard), ^{19}F NMR (376 MHz, benzotrifluoride: δ: -64.0 ppm as an external standard) and ^{77}Se NMR (76 MHz, diphenyl diselenide: δ: 463.15 ppm as external standard) spectra were recorded on JEOL ECZ-400S spectrometers in $CDCl_3$. Mass spectra were obtained on a JEOL JMP-DX300 instrument (70 eV, 300 mA). IR spectra were recorded on a Shimadzu FTIR-8400S spectrophotometer and reported in terms of frequency of absorption (cm^{-1}). Only selected IR bands are reported. UV–vis spectra were recorded at room temperature on a HITACHI U-2800A spectrophotometer and fluorescence spectra on a JASCO FP-8300 luminescence spectrometer. Chromatographic separations were carried out using Silica Gel 60N (Kanto Chemical Co., Inc.) under the solvent system stated. Thin-layer chromatography (TLC) was performed using Merck Pre-coated TLC plates (silica gel 60 F254). 1-Phenylbenzimidazole (**11**)[1] and di-*p*-tolyl diselenide (**12b**)[2] were prepared according to the published procedure. Other reagents were purchased from Wako Pure Chemical Industries, Kanto Chemical Co., Inc., and Tokyo Chemical Industry Co., Ltd.

2. Experimental details and characterization data

General procedure for the synthesis of 1-(2-bromoaryl)benzimidazoles:

Benzimidazole (5 mmol), 1-bromo-2-fluorobenzene derivatives (15 mmol, 2 equiv) and tripottasium phosphate (5.31 g, 25 mmol, 5 equiv) were dissolved in DMF (30 mL). The mixture was stirred at 150 °C. The reaction time was determined by monitoring with TLC. The reaction mixture was diluted with CH_2Cl_2 (50 mL) and water (50 mL). The phases were separated, and the aqueous layer was extracted with CH_2Cl_2 (2 × 30 mL). The combined organic layers were washed with water (3 × 50 mL), dried with $MgSO_4$, and concentrated under reduced pressure. The residue was purified by silica gel chromatography (*n*-hexane/AcOEt).

1-(2-Bromophenyl)benzimidazole (**1a**)[1]**:** Yield 2.76 g (99%); colorless prism; mp 93.5-95 °C (from *n*-hexane/CH_2Cl_2); R_f = 0.5 (*n*-hexane/EtOAc = 1:2); ^{1}H NMR (400 MHz, $CDCl_3$) δ = 7.19 (1H, dd, J = 6.8, 1.9 Hz, Ar-H), 7.26-7.53 (5H, m, Ar-H), 7.81 (1H, dd, J = 7.9, 1.1 Hz, Ar-H), 7.90 (1H, dd, J = 6.4, 1.5 Hz, Ar-H), 8.04 (1H, s, Ar-H); ^{13}C NMR (100 MHz, $CDCl_3$) δ = 110.5 (CH), 120.4 (CH), 121.4 (C), 122.7 (CH), 123.6 (CH), 128.6 (CH),

129.0 (CH), 130.5 (CH), 134.1 (CH), 134.2 (C), 135.1 (C), 142.9 (CH), 143.1 (C); LRMS (EI) *m/z* 272 (M^+, 100), 193 (94), 166 (40), 140 (18), 75 (20); HRMS: *m/z* $[M]^+$ calcd for $C_{13}H_9BrN_2$: 271.9949. Found: 271.9944.

1-(2-Bromo-5-methoxyphenyl)benzimidazole (**1b**)**:** Yield 929 mg (31%); colorless prism; mp 131-132 °C (from *n*-hexane/CH_2Cl_2); R_f = 0.2 (*n*-hexane/EtOAc = 3:2); ^{1}H NMR (400 MHz, $CDCl_3$) δ = 3.83 (3H, s, CH_3), 6.95-6.99 (2H, m, Ar-H), 7.23 (1H, dd, J = 5.5, 1.8 Hz, Ar-H), 7.30-7.37 (2H, m, Ar-H), 7.67 (1H, d, J = 8.7 Hz, Ar-H), 7.89 (1H, dd, J = 6.9, 1.4 Hz, Ar-H), 8.05 (1H, s, Ar-H); ^{13}C NMR (100 MHz, $CDCl_3$) δ = 55.8 (CH_3), 110.6 (CH), 111.3 (C), 114.5 (CH), 116.6 (CH), 120.4 (CH), 122.7 (CH), 123.7 (CH), 134.1 (C), 134.4 (CH), 135.7 (C), 142.9 (CH), 143.1 (C), 159.7 (C); ; IR (KBr); ν = 3078, 1597, 1497, 1217, 735 cm^{-1}; LRMS (EI) *m/z* 304 ($[M+2]^+$, 98), 302 (M^+, 100), 223 (93), 179 (25); HRMS: *m/z* $[M]^+$ calcd for $C_{14}H_{11}BrN_2O$: 302.0055. Found: 302.0050.

1-(2-Bromo-5-methylphenyl)benzimidazole (**1c**)**:** Yield 2.75 g (96%); colorless plate; mp 91-93 °C (from *n*-hexane/CH_2Cl_2); R_f = 0.3 (*n*-hexane/EtOAc = 3:2); ^{1}H NMR (400 MHz, $CDCl_3$) δ = 2.39 (3H, s, CH_3), 7.17-7.21 (2H, m, Ar-H), 7.24 (1H, d, J = 2.7 Hz, Ar-H), 7.29 (1H, td, J = 7.3, 1.9 Hz, Ar-H), 7.32 (1H, td, J = 7.3, 1.9 Hz, Ar-H), 7.65 (1H, d, J = 8.2 Hz, Ar-H), 7.87 (1H, d, J = 7.3 Hz, Ar-H), 8.01 (1H, s, Ar-H); ^{13}C NMR (100 MHz, $CDCl_3$) δ = 20.8 (CH_3), 110.6 (CH), 117.8 (C), 120.4 (CH), 122.6 (CH), 123.6 (CH), 129.6 (CH), 131.4 (CH), 133.7 (CH), 134.3 (C), 134.8 (C), 139.1 (C), 142.9 (CH), 143.2 (C); IR (KBr); ν = 3055, 1495, 1288, 1209, 752 cm^{-1}; LRMS (EI) *m/z* 288 ($[M+2]^+$, 98), 286 (M^+, 100), 207 (97), 192 (63); HRMS: *m/z* $[M]^+$ calcd for $C_{14}H_{11}BrN_2$: 286.0106. Found: 286.0105.

1-(2,5-Dibromophenyl)benzimidazole (**1d**)**:** Yield 3.11 g (92%); pale red plate; mp 123.5-125 °C (from *n*-hexane/CH_2Cl_2); R_f = 0.5 (*n*-hexane/EtOAc = 3:2); ^{1}H NMR (400 MHz, $CDCl_3$) δ = 7.21 (1H, d, J = 7.8 Hz, Ar-H), 7.31-7.38 (2H, m, Ar-H), 7.54 (1H, dd, J = 8.7, 2.2 Hz, Ar-H), 7.62 (1H, d, J = 2.2 Hz, Ar-H), 7.68 (1H, d, J = 8.2 Hz, Ar-H), 7.89 (1H, dd, J = 6.4, 1.3 Hz, Ar-H), 8.03 (1H, s, Ar-H); ^{13}C NMR (100 MHz, $CDCl_3$) δ = 110.5 (CH), 120.3 (C), 120.7 (CH), 121.9 (C), 123.1 (CH), 124.1 (CH), 132.1 (CH), 133.8 (CH), 134.1 (C), 135.3 (CH), 136.5 (C), 142.6 (CH), 143.3 (C); IR (KBr); ν = 3049, 1489, 1229, 993, 735 cm^{-1}; LRMS (EI) *m/z* 352 (M^+, 100), 192 (80), 271 (35); HRMS: *m/z* $[M]^+$ calcd for $C_{13}H_8Br_2N_2$: 349.9054. Found: 349.9056.

1-(2-Bromo-5-chlorophenyl)benzimidazole (**1e**)**:** Yield 1.43 g (93%); pale red plate; mp 94-96.5 °C (from *n*-hexane/CH_2Cl_2); R_f = 0.4 (*n*-hexane/EtOAc = 3:2); ^{1}H NMR (400 MHz, $CDCl_3$) δ = 7.22 (1H, d, J = 8.5 Hz, Ar-H), 7.31-7.39 (2H, m, Ar-H), 7.41 (1H, dd, J = 8.2, 2.3 Hz, Ar-H), 7.48 (1H, d, J = 2.3 Hz, Ar-H), 7.76 (1H, d, J = 8.5

Hz, Ar-H), 7.89 (1H, d, J = 6.6 Hz, Ar-H), 8.04 (1H, s, Ar-H); ^{13}C NMR (100 MHz, $CDCl_3$) δ = 110.4 (CH), 119.4 (C), 120.7 (CH), 123.0 (CH), 124.0 (CH), 129.2 (CH), 130.7 (CH), 134.0 (C), 134.4 (C), 135.0 (CH), 136.2 (C), 142.5 (CH), 143.2 (C); IR (KBr); ν = 3051, 1489, 1230, 737 cm^{-1}; LRMS (EI) m/z 308 ([M+2]$^+$, 100), 306 ([M]$^+$, 90), 192 (97), 227 (68); HRMS: m/z [M]$^+$ calcd for $C_{13}H_8BrClN_2$: 305.9559. Found: 305.9562.

1-(2-Bromo-5-trifluoromethylphenyl)benzimidazole (**1f**)**:** Yield 3.15 g (92%); colorless prism; mp 96-97.5 °C (from n-hexane/CH_2Cl_2); R_f = 0.4 (n-hexane/EtOAc = 3:2); ^{1}H NMR (400 MHz, $CDCl_3$) δ = 7.19 (1H, d, J = 8.3 Hz, Ar-H), 7.35 (1H, t, J = 7.3 Hz, Ar-H), 7.38 (1H, t, J = 6.4 Hz, Ar-H), 7.68 (1H, d, J = 6.9 Hz, Ar-H), 7.74 (1H, s, Ar-H), 7.91 (1H, d, J = 7.8 Hz, Ar-H), 7.99 (1H, d, J = 8.3 Hz, Ar-H), 8.07 (1H, s, Ar-H); ^{13}C NMR (100 MHz, $CDCl_3$) δ = 110.2 (CH), 120.7 (CH), 123.0 (q, J = 272 Hz, CF_3), 123.2 (CH), 124.1 (CH), 125.6 (C), 126.0 (q, J = 3.9 Hz, CH), 127.2 (q, J = 3.8 Hz, CH), 131.5 (q, J = 34 Hz, C), 133.9 (C), 135.1 (CH), 136.0 (C), 142.4 (CH), 143.2 (C); ^{19}F NMR (376 MHz, $CDCl_3$) δ = -62.6; IR (KBr); ν = 3016, 1497, 1290, 1084, 741 cm^{-1}; LRMS (EI) m/z 340 (M$^+$, 100), 261 (35), 192 (29); HRMS: m/z [M]$^+$ calcd for $C_{14}H_8BrF_3N_2$: 339.9823. Found: 339.9821.

1-(2-Bromophenyl)-5,6-dimethylbenzimidazole (**1g**)**:** Yield 1.49 g (>99%); colorless plate; mp 73-75 °C (from n-hexane/CH_2Cl_2); R_f = 0.2 (n-hexane/EtOAc = 3:2); ^{1}H NMR (400 MHz, $CDCl_3$) δ = 2.34 (3H, s, CH_3), 2.40 (3H, s, CH_3), 6.96 (1H, s, Ar-H), 7.37-7.44 (2H, m, Ar-H), 7.50 (1H, t, J = 7.8 Hz, Ar-H), 7.64 (1H, s, Ar-H), 7.81 (1H, d, J = 6.8 Hz, Ar-H), 7.93 (1H, s, Ar-H); ^{13}C NMR (100 MHz, $CDCl_3$) δ = 20.2 (CH_3), 20.5 (CH_3), 110.5 (CH), 120.4 (CH), 121.4 (C), 128.6 (CH), 129.1 (CH), 130.4 (CH), 131.6 (C), 132.8 (C), 132.9 (C), 134.1 (CH), 135.4 (C), 141.7 (C), 142.2 (CH); IR (KBr); ν = 3090, 1497, 1223, 841, 772 cm^{-1}; LRMS (EI) m/z 302 ([M+2]$^+$, 99) 300 (M$^+$, 100), 285 (40), 205 (32); HRMS: m/z [M]$^+$ calcd for $C_{15}H_{13}BrN_2$: 300.0262. Found: 300.0260.

1-(2-Bromophenyl)-5,6-dichlorobenzimidazole (**1h**)**:** Yield 1.05 g (61%); colorless plate; mp 110-113 °C (from Benzene); R_f = 0.4 (n-hexane/EtOAc = 3:2); ^{1}H NMR (400 MHz, $CDCl_3$) δ = 7.29 (1H, s, Ar-H), 7.44 (1H, d, J = 8.2 Hz, Ar-H), 7.45 (1H, td, J = 7.8, 1.9 Hz, Ar-H), 7.55 (1H, td, J = 7.8, 3.0 Hz, Ar-H), 7.83 (1H, d, J = 5.3 Hz, Ar-H), 7.98 (1H, s, Ar-H), 8.04 (1H, s, Ar-H); ^{13}C NMR (100 MHz, $CDCl_3$) δ = 112.1 (CH), 121.5 (C), 121.8 (CH), 127.2 (C), 128.1 (C), 129.0 (CH), 129.1 (CH), 131.3 (CH), 133.6 (C), 134.4 (C), 134.5 (CH), 142.6 (C), 144.8 (CH); IR (KBr); ν = 3096, 1493, 1236, 1096, 766 cm^{-1}; LRMS (EI) m/z 342 ([M+2]$^+$, 100), 226 (50), 261 (30); HRMS: m/z [M]$^+$ calcd for $C_{13}H_7BrCl_2N_2$: 339.9170. Found: 39.9171.

1-(2-Bromophenyl)imidazole (**1i**)[3]**:** Yield 793 mg (36%); orange oil; R_f = 0.5 (EtOAc); ^{1}H NMR (400 MHz, $CDCl_3$)

δ = 7.14 (1H, s, Ar-H), 7.21 (1H, s, Ar-H), 7.31-7.35 (2H, m, Ar-H), 7.45 (1H, td, J = 7.6 Hz, Ar-H), 7.68 (1H, s, Ar-H), 7.74 (1H, d, J = 6.8 Hz, Ar-H); ^{13}C NMR (100 MHz, $CDCl_3$) δ = 119.9 (C), 120.5 (CH), 128.0 (CH), 128.4 (CH), 129.3 (CH), 130.1 (CH), 133.9 (CH), 136.7 (CH), 137.5 (CH); IR (KBr); v = 3111, 1499, 1061, 760 cm^{-1}; LRMS (EI) m/z 222 (M^+, 100), 195 (18), 155 (16), 116, (83), 89 (33); HRMS: m/z $[M]^+$ calcd for $C_9H_7BrN_2$: 221.9793. Found: 221.9791.

General procedure for the synthesis of benzimidazo[2,1-*b*]benzoselenoazoles:

1-(2-Bromoaryl)benzimidazoles **1** (0.5 mmol), selenium powder (79 mg, 1.0 mmol, 2 equiv), and cesium carbonate (326 mg, 1.0 mmol, 2 equiv) were dissolved in DMF (1 mL) under Ar atmosphere. The mixture was stirred at 150 °C for 24 h. The reaction mixture was diluted with CH_2Cl_2 (15 mL) and water (15 mL). The phases were separated, and the aqueous layer was extracted with CH_2Cl_2 (2 × 10 mL). The combined organic layers were washed with water (2 × 20 mL), dried with $MgSO_4$, and concentrated under reduced pressure. The residue was purified by silica gel chromatography (*n*-hexane/AcOEt).

Benzimidazo[2,1-*b*]benzoselenoazole (**2a**)**:** Yield 125 mg (93%); yellow needle; mp 152.5-154 °C (from *n*-hexane/CH_2Cl_2); R_f = 0.5 (*n*-hexane/EtOAc = 3:2); ^{1}H NMR (400 MHz, $CDCl_3$) δ = 7.27 (1H, t, J = 7.3 Hz, Ar-H), 7.33-7.40 (2H, m, Ar-H), 7.51 (1H, t, J = 7.3 Hz, Ar-H), 7.73 (1H, d, J = 7.8 Hz, Ar-H), 7.81 (1H, dd, J = 7.3, 1.5 Hz, Ar-H), 7.96 (2H, d, J = 8.3 Hz, Ar-H); ^{13}C NMR (100 MHz, $CDCl_3$) δ = 110.7 (CH), 113.3 (CH), 119.2 (CH), 122.0 (CH), 123.3 (CH), 124.5 (CH), 127.0 (CH), 127.2 (CH), 127.3 (C), 131.6 (C), 134.6 (C), 148.0 (C), 152.7 (C); IR (KBr); v = 1476, 1445, 1238, 731 cm^{-1}; LRMS (EI) m/z 272 (M^+, 100), 192 (13); HRMS: m/z $[M]^+$ calcd for $C_{13}H_8N_2Se$: 271.9853. Found: 271.9850.

2-Methylbenzimidazo[2,1-*b*]benzoselenoazole (**4**)**:** Yield 101 mg (70%); colorless needle; mp 219-221.5 °C (from *n*-hexane/CH_2Cl_2); R_f = 0.6 (*n*-hexane/EtOAc = 3:2); ^{1}H NMR (400 MHz, $CDCl_3$) δ = 2.52 (3H, s, CH_3), 7.09 (1H, d, J = 7.7 Hz, Ar-H), 7.32-7.40 (2H, m, Ar-H), 7.57 (1H, d, J = 7.8 Hz, Ar-H), 7.76 (1H, s, Ar-H), 7.80 (1H, d, J = 7.1 Hz, Ar-H), 7.96 (1H, dd, J = 7.1, 1.8 Hz, Ar-H); ^{13}C NMR (100 MHz, $CDCl_3$) δ = 21.7 (CH_3), 110.7 (CH), 114.0 (CH), 119.1 (CH), 121.8 (CH), 123.2 (CH), 123.7 (C), 125.5 (CH), 126.8 (CH), 131.6 (C), 134.7 (C), 137.4 (C), 148.1 (C), 153.1 (C); ^{77}Se NMR (76 MHz, $CDCl_3$) δ = 348.0; IR (KBr); v = 1559, 1487, 1244, 733 cm^{-1}; LRMS (EI) m/z 286 (M^+, 100), 205 (19), 78 (18); HRMS: m/z $[M]^+$ calcd for $C_{14}H_{10}N_2Se$: 286.0009. Found: 286.0006.

2-Chlorobenzimidazo[2,1-*b*]benzoselenoazole (**6**)**:** Yield 76 mg (49%); pale yellow needle; mp 264.5-267 °C (from chloroform); R_f = 0.5 (*n*-hexane/EtOAc = 3:2); ^{1}H NMR (400 MHz, $CDCl_3$) δ = 7.35-7.42 (2H, m, Ar-H), 7.46 (1H, dd, J = 8.2, 1.8 Hz, Ar-H), 7.74 (1H, dd, J = 6.8, 1.8 Hz, Ar-H), 8.18 (1H, d, J = 8.7 Hz, Ar-H), 8.39 (1H, d, J = 1.8 Hz, Ar-H), 8.46 (1H, dd, J = 6.4, 1.8 Hz, Ar-H); ^{13}C NMR (100 MHz, $CDCl_3$) δ = 110.7 (CH), 113.7 (CH), 119.4 (CH), 122.3 (CH), 123.7 (CH), 124.6 (CH), 125.4 (C), 127.9 (CH), 131.4 (C), 133.2 (C), 135.3 (C), 148.1 (C), 152.8 (C); ^{77}Se NMR (76 MHz, $CDCl_3$) δ = 356.0; IR (KBr); ν = 1487, 1449, 1221, 731, 550 cm^{-1}; LRMS (EI) *m/z* 306 (M^+, 100), 191 (10), 153 (10); HRMS: *m/z* $[M]^+$ calcd for $C_{13}H_7ClN_2Se$: 305.9463. Found: 305.9466.

2-Trifluoromethylbenzimidazo[2,1-*b*]benzoselenoazole (**7**)**:** Yield 107 mg (63%); colorless needle; mp 207-208 °C (from *n*-hexane/CH_2Cl_2); R_f = 0.5 (*n*-hexane/EtOAc = 3:2); ^{1}H NMR (400 MHz, $CDCl_3$) δ = 7.41-7.47 (2H, m, Ar-H), 7.58 (1H, d, J = 8.2 Hz, Ar-H), 7.83-7.87 (1H, m, Ar-H), 7.91 (1H, d, J = 8.2 Hz, Ar-H), 8.00-8.02 (1H, m, Ar-H), 8.18 (1H, s, Ar-H); ^{13}C NMR (100 MHz, $CDCl_3$) δ =109.8 (CH), 110.7 (CH), 119.6 (CH), 121.0 (CH), 122.6 (CH), 123.8 (q, J = 272 Hz, CF_3), 123.9 (CH), 127.7 (CH), 129.8 (q, J = 33 Hz, C), 131.4 (C), 131.9 (C), 134.8 (C), 148.1 (C), 152.3 (C); ^{19}F NMR (376 MHz, $CDCl_3$) δ = -63.27; ^{77}Se NMR (76 MHz, $CDCl_3$) δ = 364.3; IR (KBr); ν = 1491, 1317, 1109, 73 cm^{-1}; LRMS (EI) *m/z* 340 (M^+, 100), 170 (6), 260 (4); HRMS: *m/z* $[M]^+$ calcd for $C_{14}H_7F_3N_2Se$: 339.9727. Found: 339.9725.

8,9-Dimethylbenzimidazo[2,1-*b*]benzoselenoazole (**8**)**:** Yield 137 mg (91%); colorless plate; mp 95-96.5 °C (from *n*-hexane/CH_2Cl_2); R_f = 0.5 (*n*-hexane/EtOAc = 3:2); ^{1}H NMR (400 MHz, $CDCl_3$) δ = 2.42 (3H, s, CH_3), 2.47 (3H, s, CH_3), 7.28 (1H, t, J = 7.8 Hz, Ar-H), 7.53 (1H, t, J = 7.1 Hz, Ar-H), 7.58 (1H, s, Ar-H), 7.73-7.75 (2H, m, Ar-H), 7.97 (1H, d, J = 6.9 Hz, Ar-H); ^{13}C NMR (100 MHz, $CDCl_3$) δ = 20.5 (CH_3), 20.7 (CH_3), 111.1 (CH), 113.2 (CH), 119.4 (CH), 124.2 (CH), 127.0 (CH), 127.3 (CH), 127.4 (C), 130.2 (C), 131.0 (C), 132.2 (C), 134.9 (C), 146.8 (C), 151.5 (C); ^{77}Se NMR (76 MHz, $CDCl_3$) δ = 350.1; IR (KBr); ν = 3057, 1487, 1244, 1153, 733 cm^{-1}; LRMS (EI) *m/z* 300 (M^+, 100), 283 (42); HRMS: *m/z* $[M]^+$ calcd for $C_{15}H_{12}N_2Se$: 300.0166. Found: 300.0163.

8,9-Dichlorobenzimidazo[2,1-*b*]benzoselenoazole (**9**)**:** Yield 74 mg (44%); colorless needle; mp 245-247.5 °C (from chloroform); R_f = 0.7 (*n*-hexane/EtOAc = 3:2); ^{1}H NMR (400 MHz, $CDCl_3$) δ = 7.34 (1H, t, J = 7.1 Hz, Ar-H), 7.56 (1H, t, J = 8.1 Hz, Ar-H), 7.77 (1H, dd, J = 7.7, 0.9 Hz, Ar-H), 7.87 (1H, s, Ar-H), 7.87 (1H, dd, J = 5.8, 0.9 Hz, Ar-H), 8.04 (1H, s, Ar-H); ^{13}C NMR (100 MHz, $CDCl_3$) δ = 111.9 (CH), 113.3 (CH), 120.2 (CH), 125.2 (CH), 125.9 (C), 127.37 (CH), 127.43 (CH), 127.5 (C), 130.5 (C), 133.9 (C), 147.2 (C), 155.0 (C); ^{77}Se NMR (76 MHz, $CDCl_3$) δ =

359.3; IR (KBr); *v* = 1559, 1541, 1506, 1456 cm^{-1}; LRMS (EI) *m/z* 340 (M^+, 100), 207 (61), 281 (31); HRMS: *m/z* $[M]^+$ calcd for $C_{13}H_6Cl_2N_2Se$: 339.9073. Found: 339.9070.

Imidazo[2,1-*b*]benzoselenoazole (**10**)**:** Yield 46 mg (21%); orange oil; R_f = 0.6 (*n*-hexane/EtOAc = 1:2); ^{1}H NMR (400 MHz, $CDCl_3$) δ = 7.26 (1H, t, *J* = 6.9 Hz, Ar-H), 7.35 (1H, s, Ar-H), 7.43 (1H, t, *J* = 6.8 Hz, Ar-H), 7.56 (1H, d, *J* = 7.3 Hz, Ar-H), 7.72 (1H, d, *J* = 7.8 Hz, Ar-H), 7.75 (1H, s, Ar-H); ^{13}C NMR (100 MHz, $CDCl_3$) δ = 113.1 (CH), 113.6 (CH), 125.0 (CH), 126.6 (CH), 127.3 (CH), 128.9 (C), 133.4 (C), 134.1 (CH), 143.5 (C); ^{77}Se NMR (76 MHz, $CDCl_3$) δ = 369.9; IR (KBr); *v* = 3015, 1485, 1444, 1321, 1116, 750, 713, 665 cm^{-1}; LRMS (EI) *m/z* 222 (M^+, 100), 142 (13); HRMS: *m/z* $[M]^+$ calcd for $C_9H_6N_2Se$: 221.9696. Found: 221.9696.

1-[2-(*p*-Tolylselanyl)phenyl]benzo[*d*]imidazole (**14**)**:** Yield 55 mg (30%); colorless prism; mp 134-136.5 °C (from *n*-hexane/CH_2Cl_2); R_f = 0.4 (*n*-hexane/EtOAc = 2:1); ^{1}H NMR (400 MHz, $CDCl_3$) δ = 2.31 (3H, s, CH_3), 7.06 (2H, d, *J* = 8.2 Hz, Ar-H), 7.17 (1H, d, *J* = 7.8 Hz, Ar-H), 7.27 (1H, d, *J* = 6.7 Hz, Ar-H), 7.30-7.35 (7H, m, Ar-H), 7.88 (1H, d, *J* = 7.8 Hz, Ar-H), 8.02 (1H, s, Ar-H); ^{13}C NMR (100 MHz, $CDCl_3$) δ = 21.1 (CH_3), 110.5 (CH), 120.2 (C), 122.5 (CH), 123.4 (C), 123.9 (CH), 127.6 (CH), 128.1 (CH), 129.8 (CH), 130.4 (CH), 132.4 (CH), 133.3 (C), 134.3 (C), 135.0 (C), 135.4 (CH), 138.8 (C), 142.9 (C), 143.1 (CH); ^{77}Se NMR (76 MHz, $CDCl_3$) δ = 383.2; IR (KBr); *v* = 1578, 1456, 1280, 1228, 810, 760, 500 cm^{-1}; LRMS (EI) *m/z* 364 (M^+, 73), 283 (44), 193 (100), 167 (33), 140 (13), 91 (24); HRMS: *m/z* $[M]^+$ calcd for $C_{20}H_{16}N_2Se$: 364.0479. Found: 364.0472.

3. X-ray crystal structure determinations of 2a

The crystal obtains *p*-hydroquinone which was contained in recrystallization solvent as stabilizer. The colorless prismatic crystal (0.100 × 0.050 × 0.050 mm^3), obtained from diisopropyl ether/hexane, was immersed in Paraton-N oil and placed in the N_2 cold stream at 100 K. Data were collected using diffractometer with CMOS detector (Bruker VENTURE) from Bruker AXS, Cu Kα: λ = 1.54178 Å). Absorption correction was performed by an empirical method implemented in SADABS.[4] Structure solution and refinement were performed by using SHELXT-2014/5[5] and SHELXL-2018/3[6].

$C_{29}H_{19}N_4OSe$, Mr = 721.67; monoclinic, space group $P2_1/c$, $Z = 4$, $D_{calc} = 1.748$ g·cm^{-3}, $a = 7.4069(13)$, $b = 20.254(4)$, $c = 15.134(3)$ Å, $\beta = 91.658(4)$°, $V = 2269.5(7)$ Å^3, 29686 observed and 4679 independent [$I > 2\sigma(I)$] reflections, 327 parameters, final $R_1 = 0.0230$, $wR_2 = 0.0620$, $S = 1.083$ [$I > 2\sigma(I)$]. CCDC 1918716.

All non-hydrogen atoms were refined anisotropically. The hydrogen atoms were refined isotropically on the calculated positions using a riding model (AFIX 148 and 43) with Uiso values constrained to 1.2/1.5 Ueq of their parent atoms.

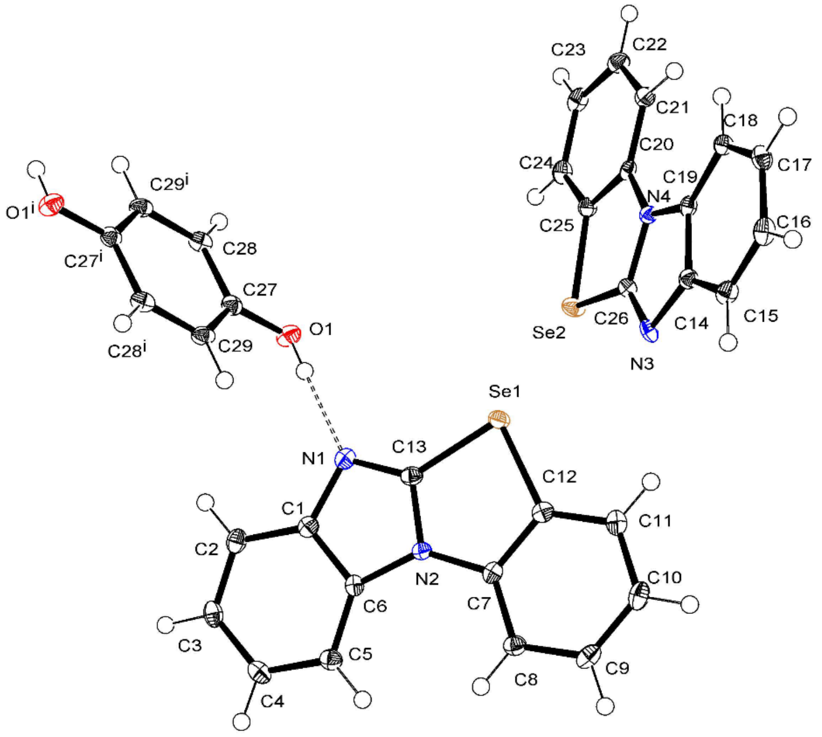

Figure S1. Ortep drawing of **2a** (50% probability)

4. Absorption spectra of benzoimidazo[2,1-*b*]benzoselenoazoles

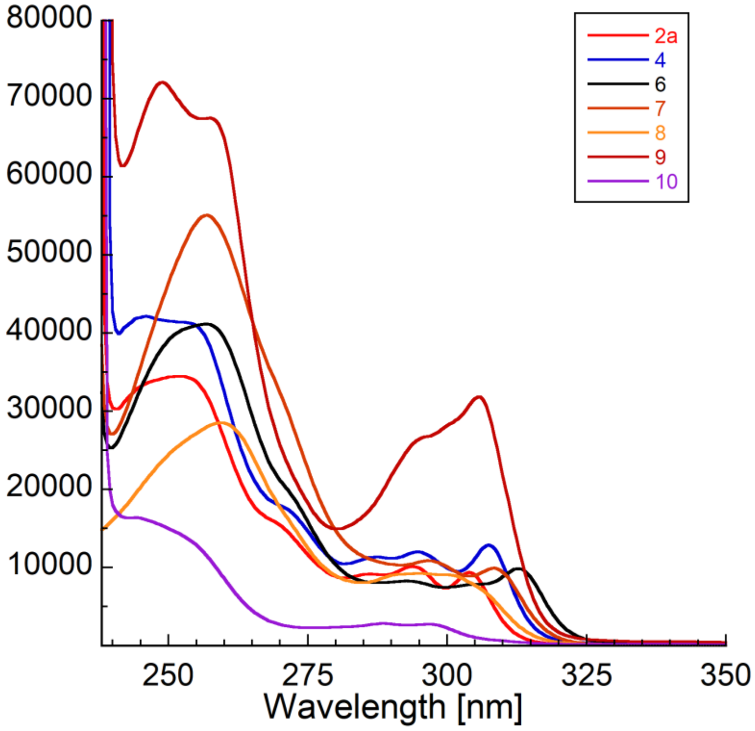

Figure S2**.** Absorption spectra of Selected compounds in $CHCl_3$.

5. References

1. Diness, F.; Fairlie, D. P. *Angew. Chem. Int. Ed.* **2012**, 51, 8012–8016.
2. Li, Z.; Ke, F.; Deng, H.; Xu, H.; Xiang, H.; Zhou, X. *Org. Biomol. Chem.* **2013**, 11, 2943–2946.
3. Schröder, K.; Enthaler, S.; Bitterlich, B.; Schulz, T.; Spannenberg, A.; Tse, M. K.; Junge, K.; Beller, M. *Chem. Eur. J.* **2008**, 15, 5471–5481.
4. Sheldrick, G. M. (1996). *SADABS*. University of Göttingen, Germany.
5. Sheldrick, G. M. *Acta. Cryst.* **2015**, *A71*, 3–8.
6. Sheldrick, G. M. *Acta. Cryst.* **2015**, *C71*, 3–8.

7. NMR spectra of new compounds

^{1}H NMR of **1a**

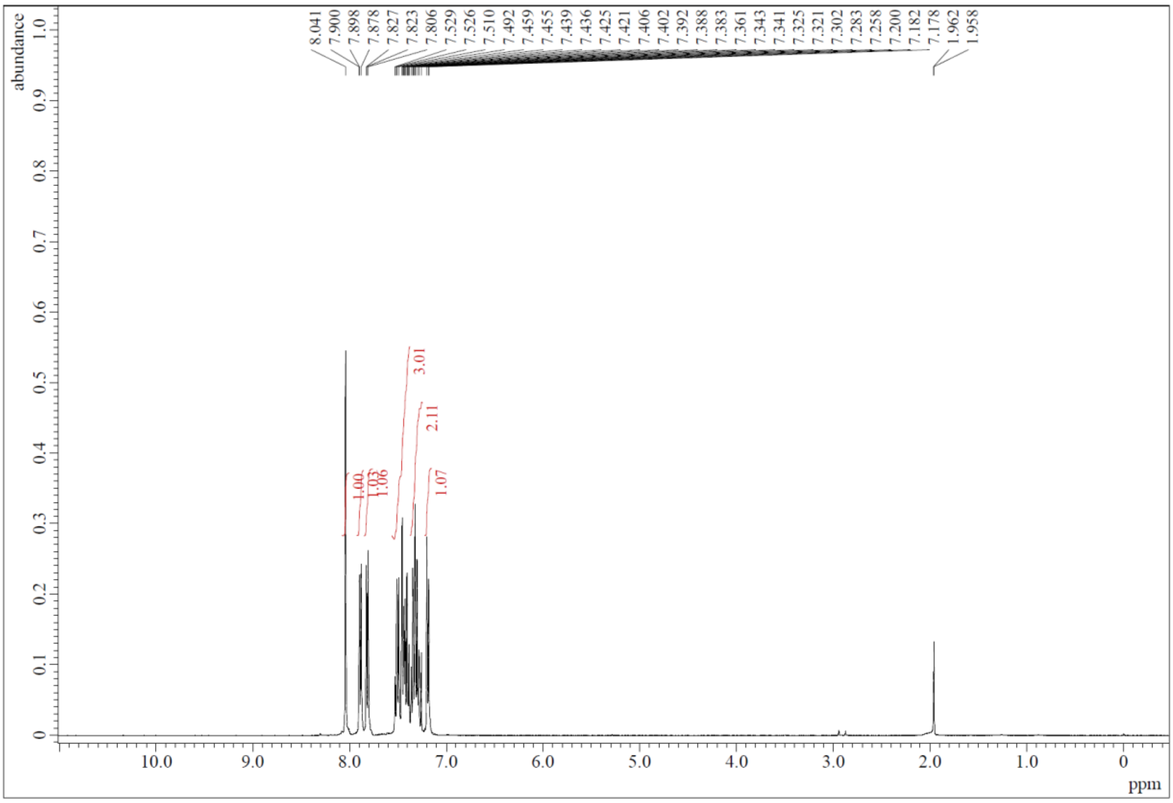

^{13}C NMR of **1a**

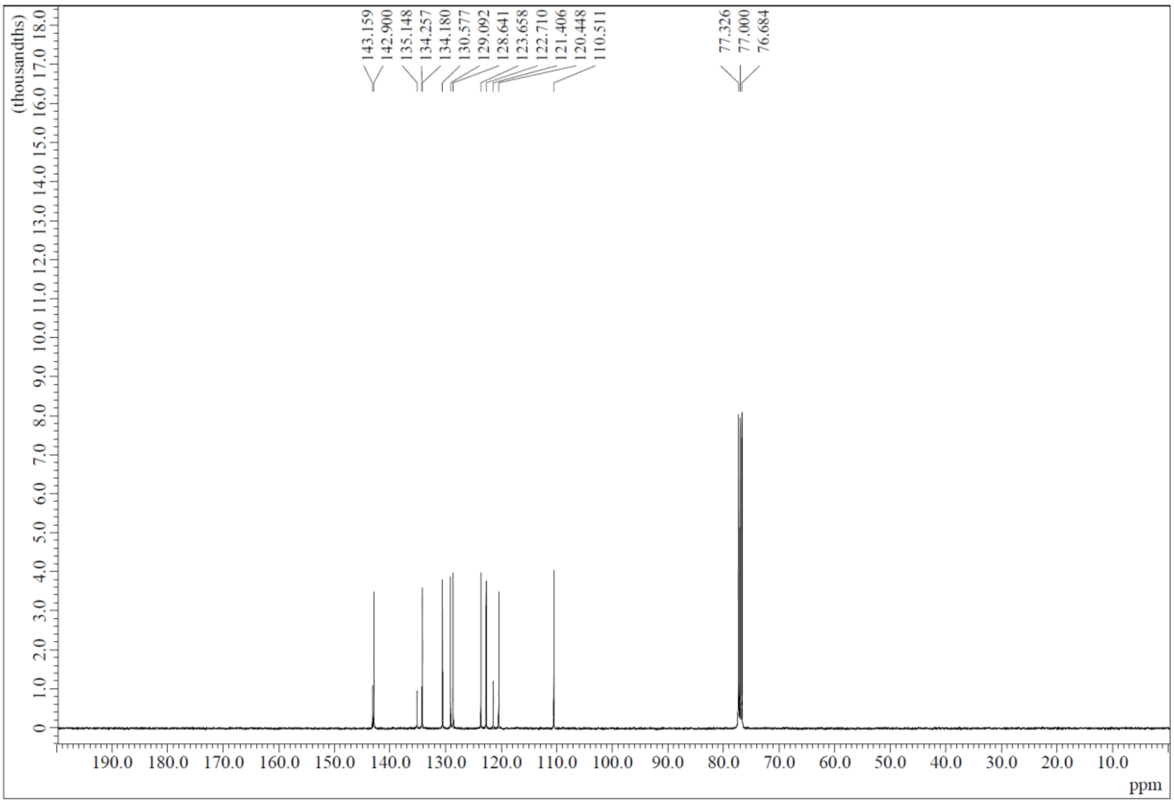

^{1}H NMR of **1b**

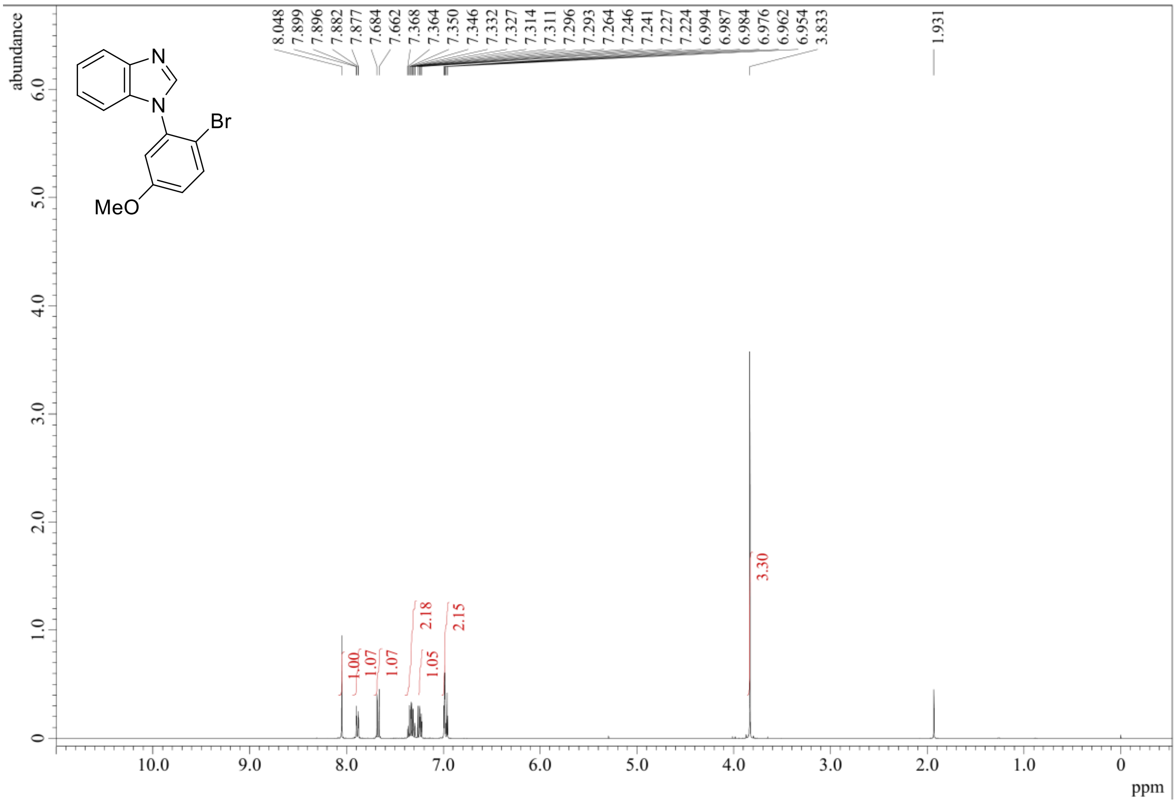

^{13}C NMR of **1b**

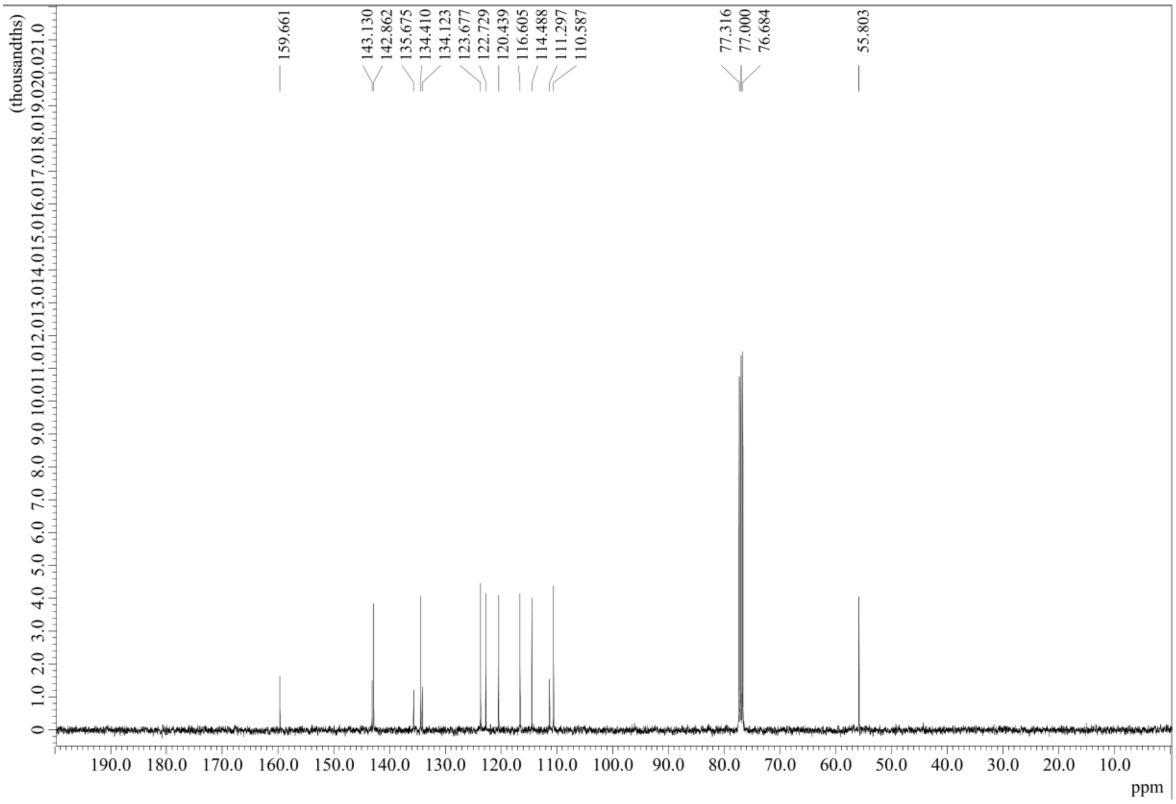

^{1}H NMR of **1c**

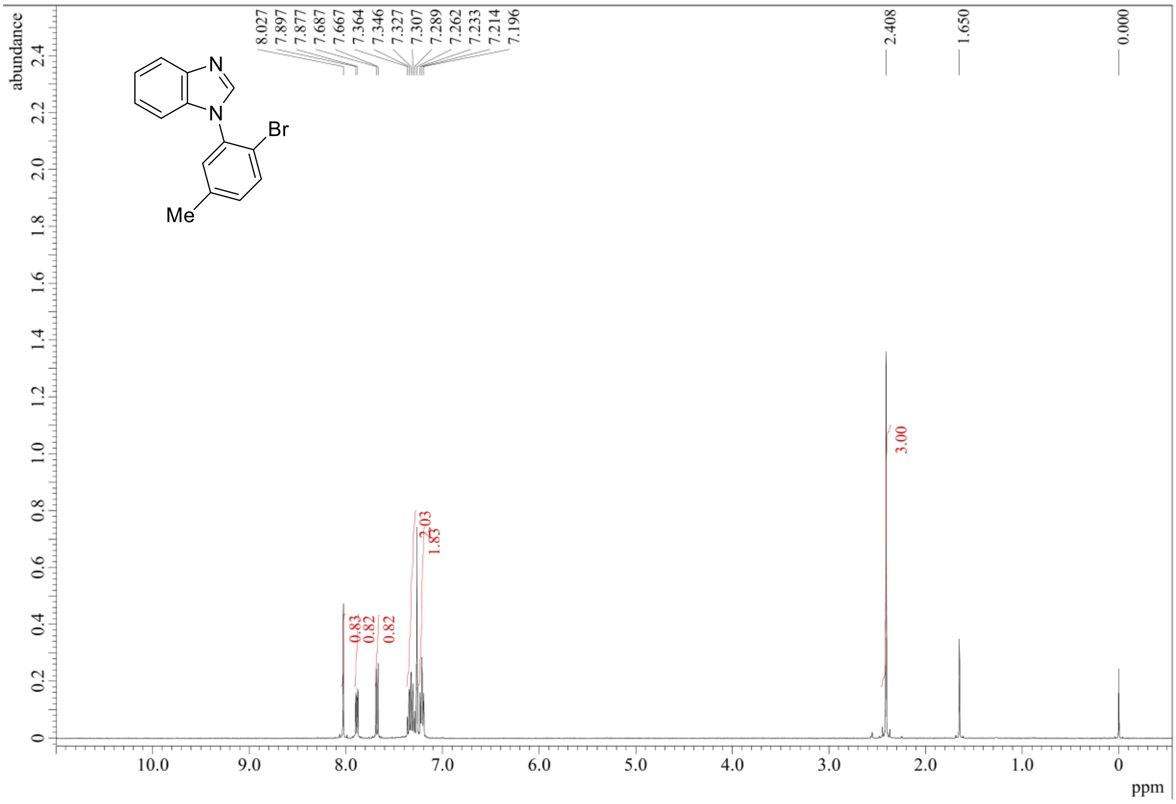

^{13}C NMR of **1c**

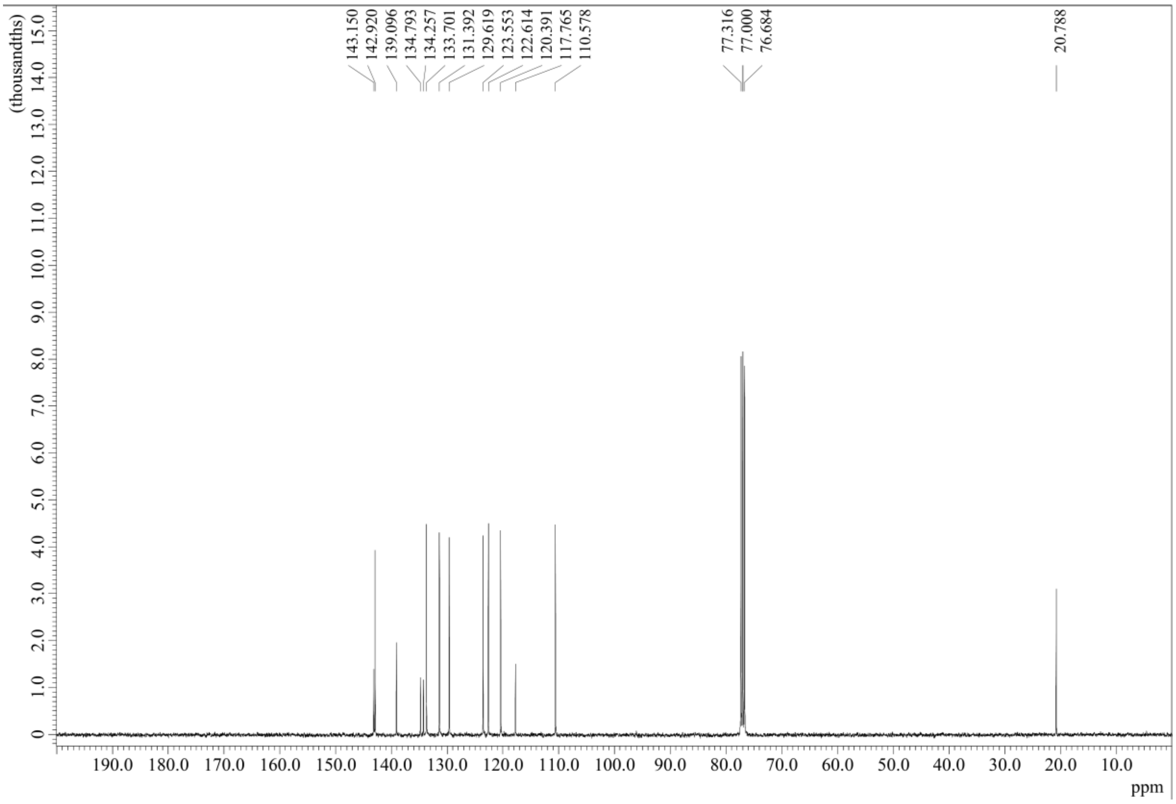

^{1}H NMR of **1d**

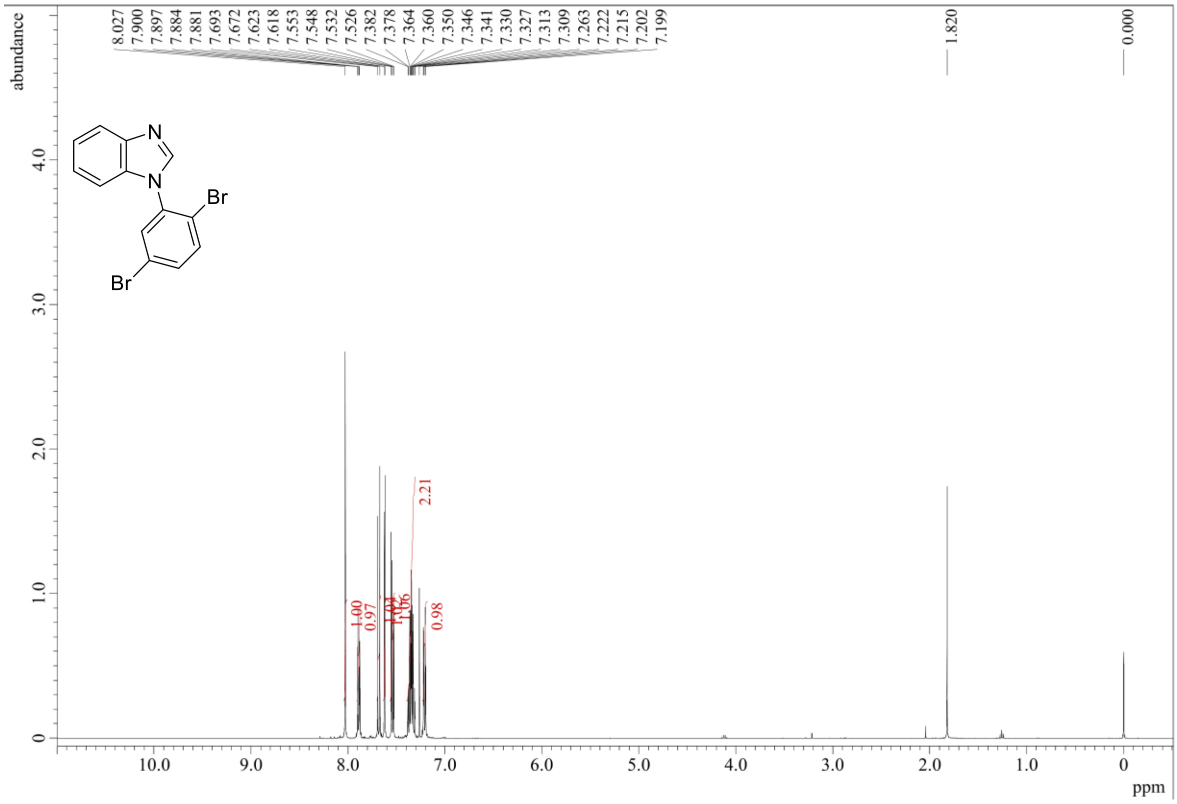

^{13}C NMR of **1d**

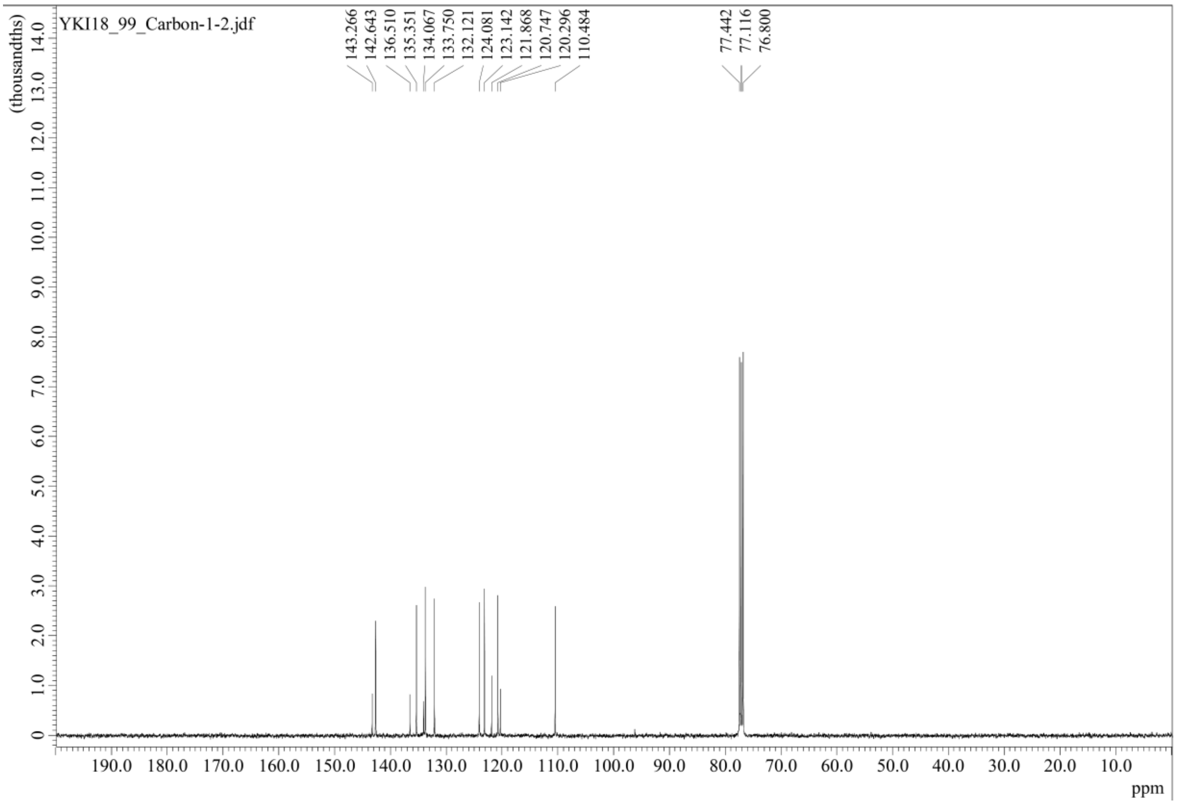

^{1}H NMR of **1e**

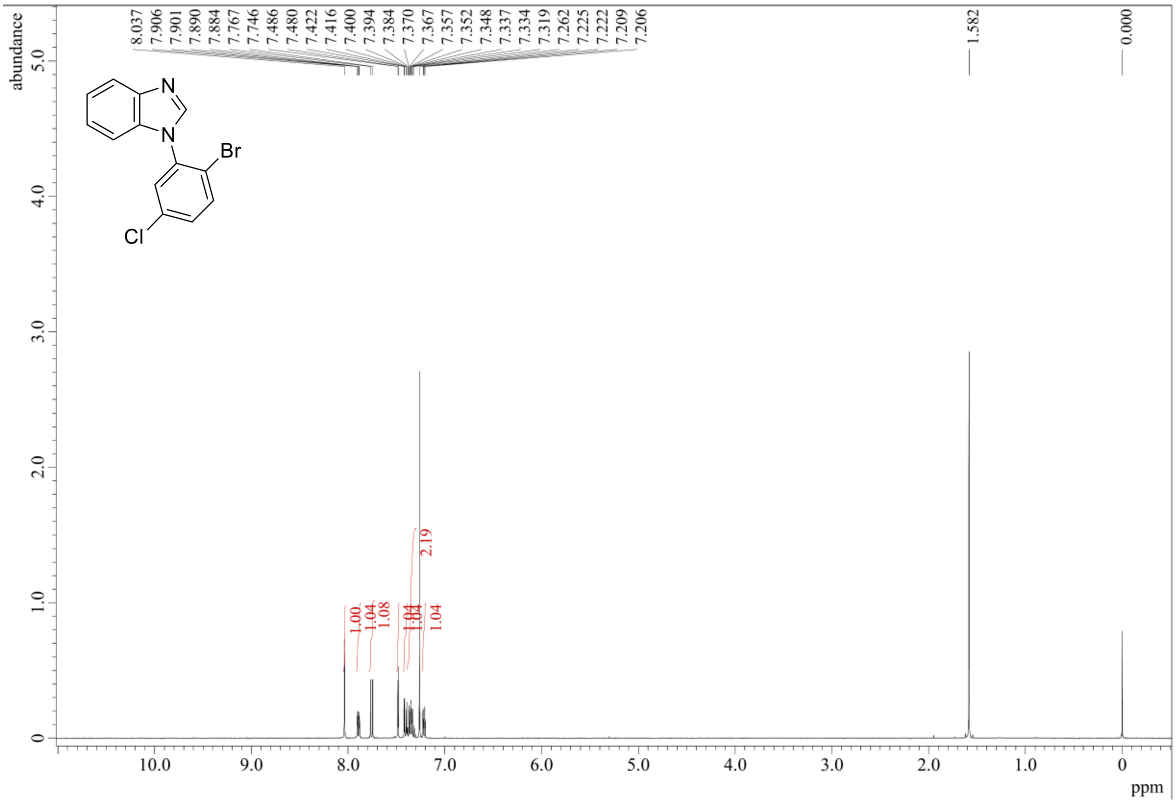

^{13}C NMR of **1e**

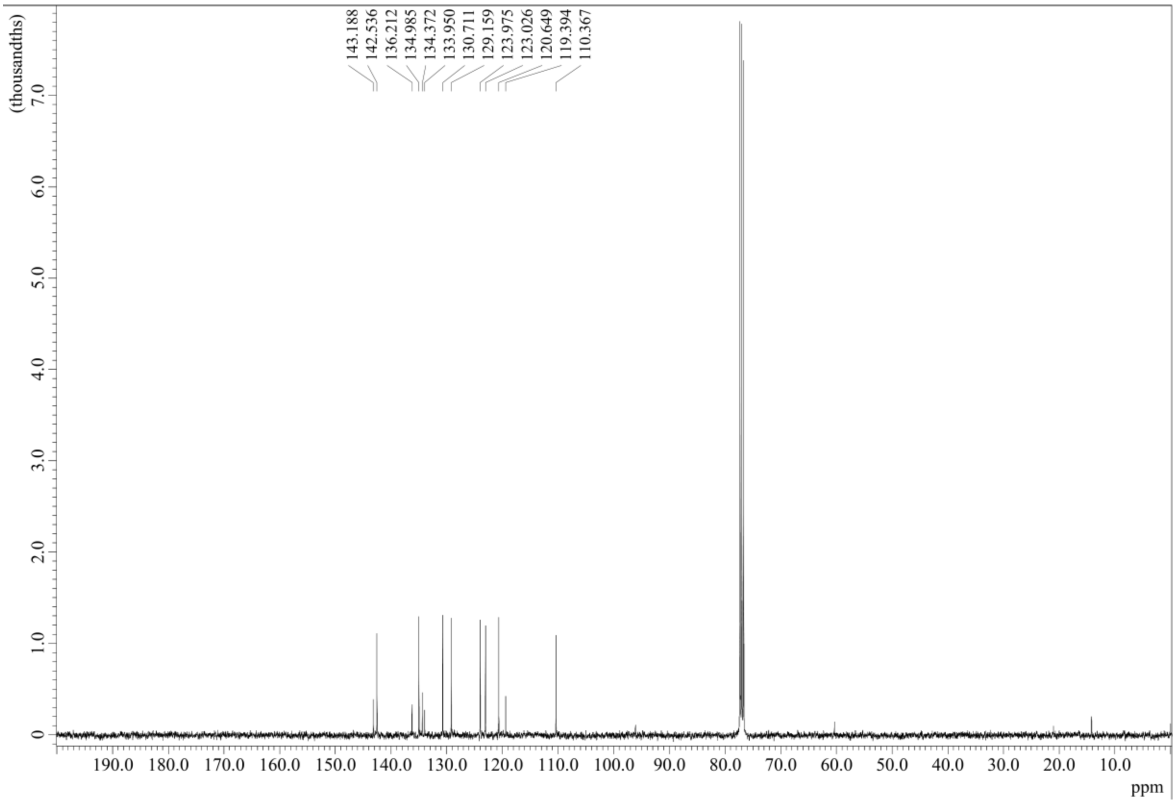

^{1}H NMR of **1f**

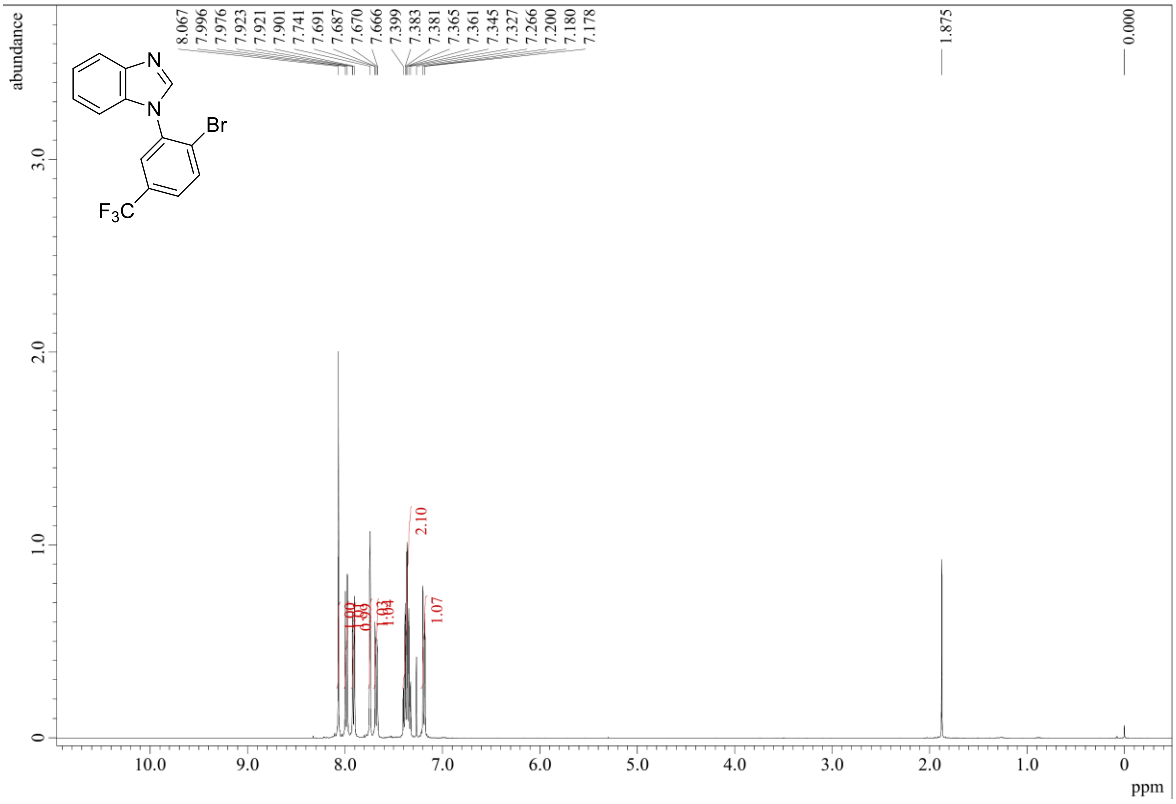

^{13}C NMR of **1f**

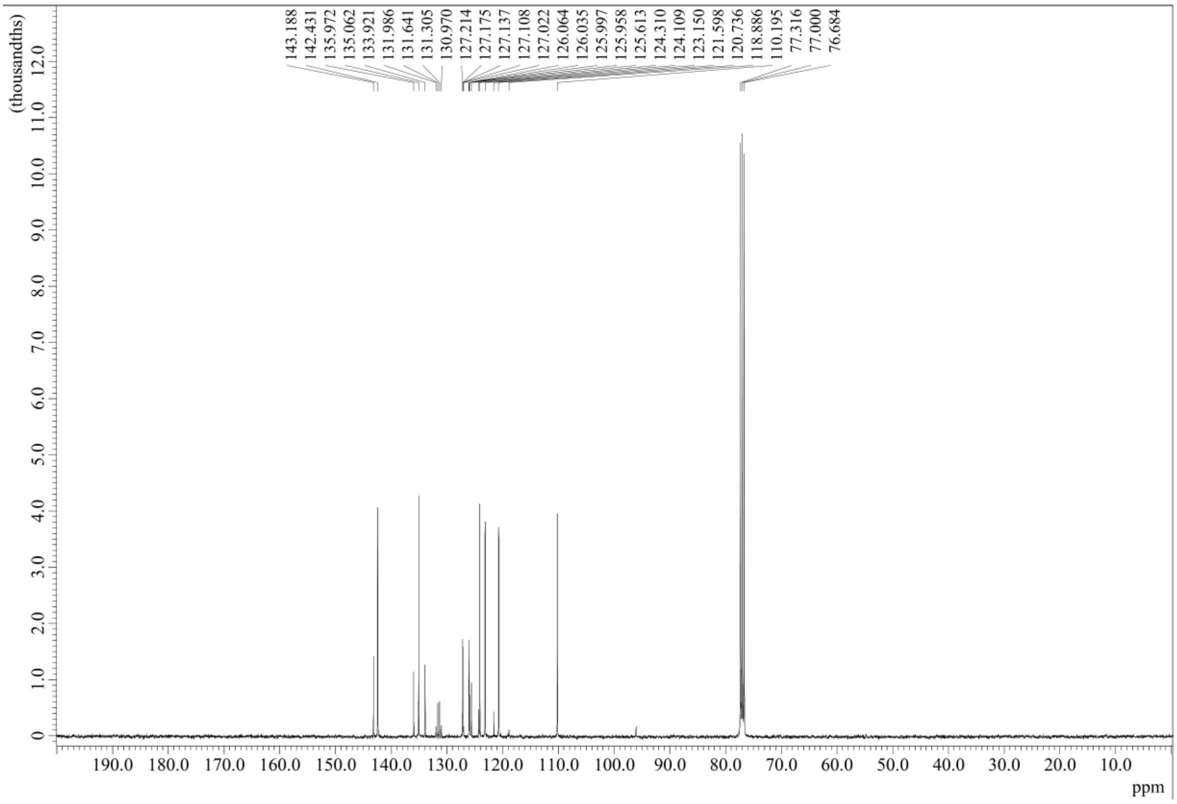

^{1}H NMR of **1g**

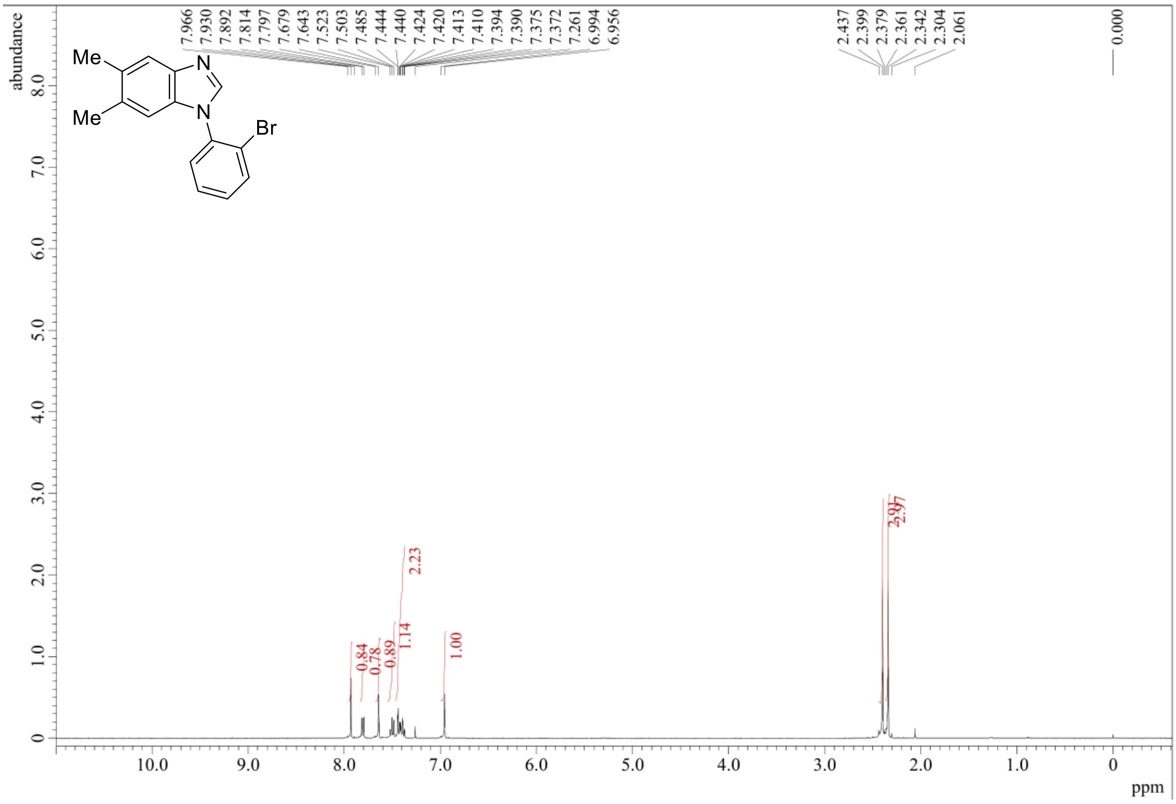

^{13}C NMR of **1g**

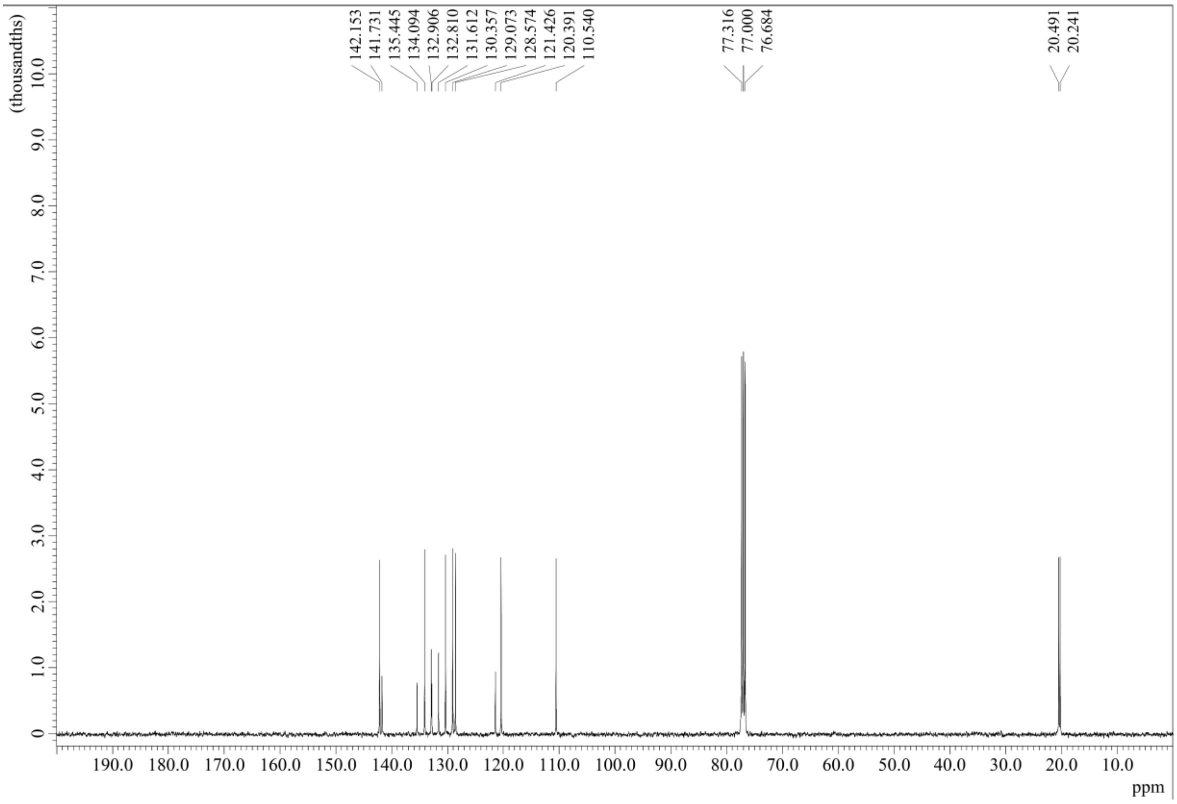

^{1}H NMR of **1h**

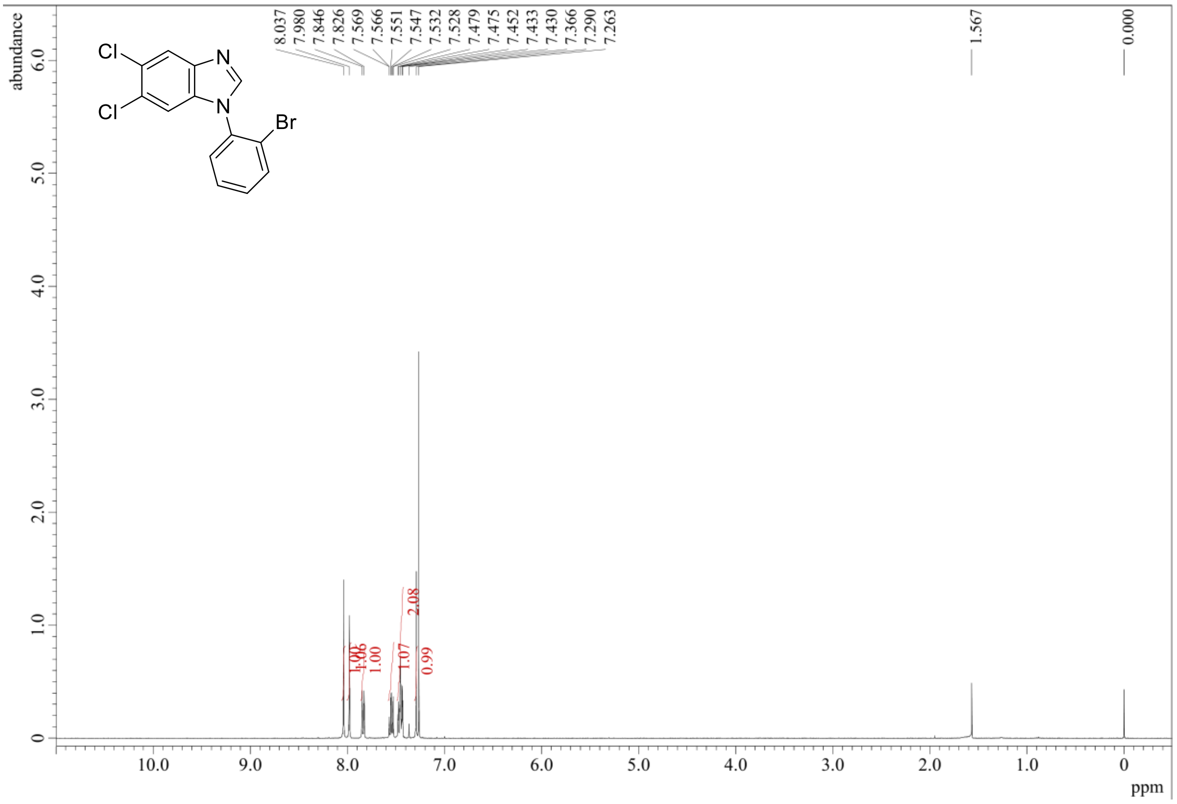

^{13}C NMR of **1h**

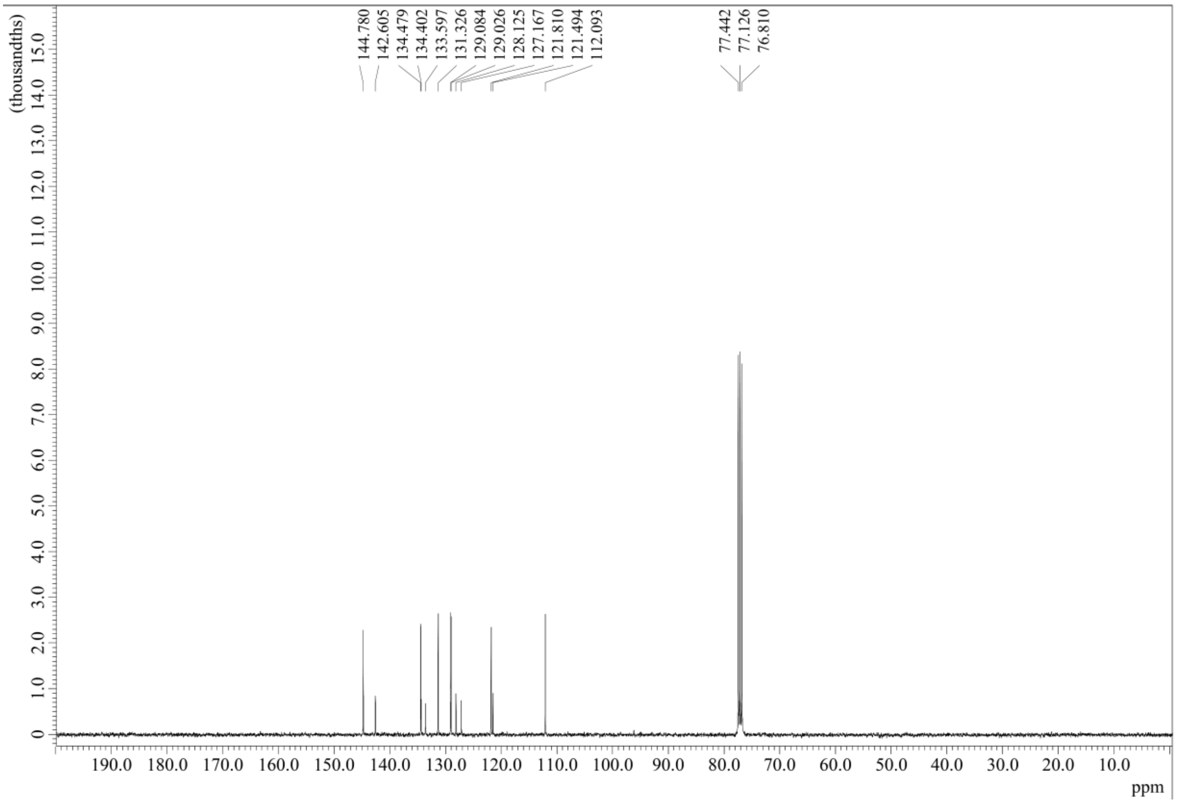

^{1}H NMR of **1i**

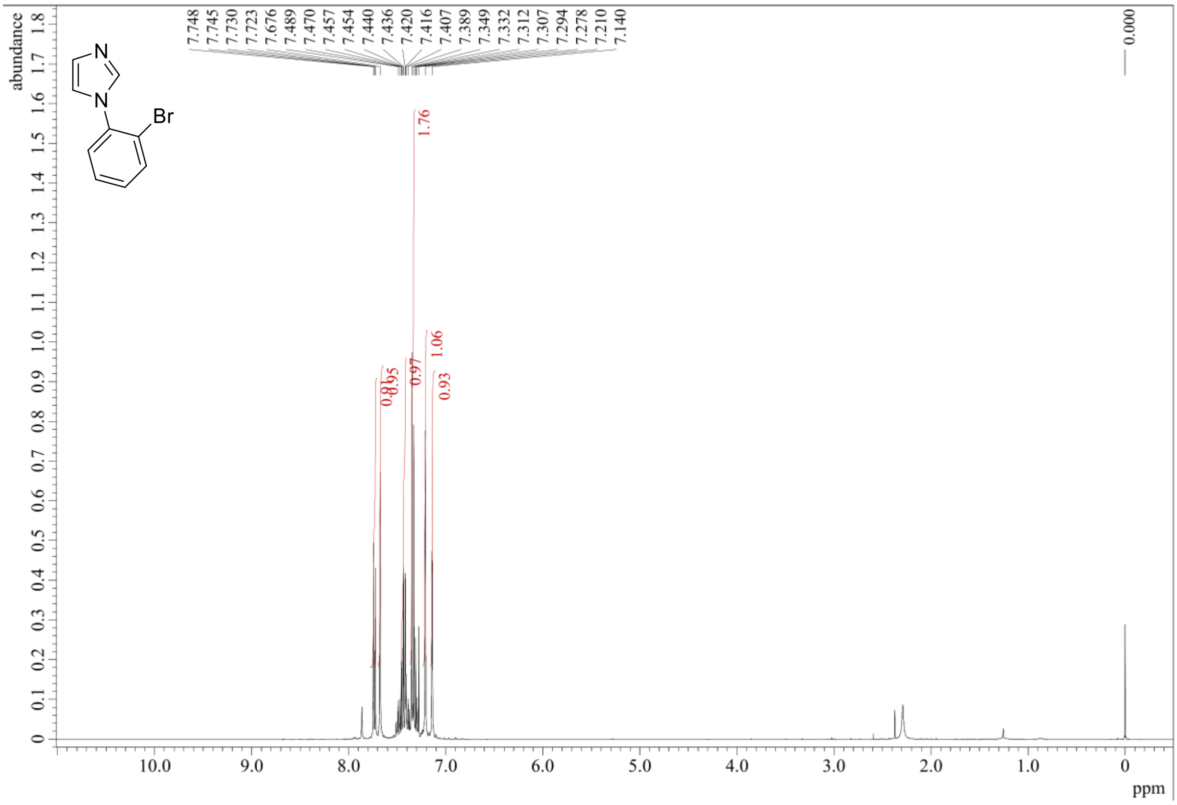

^{13}C NMR of **1i**

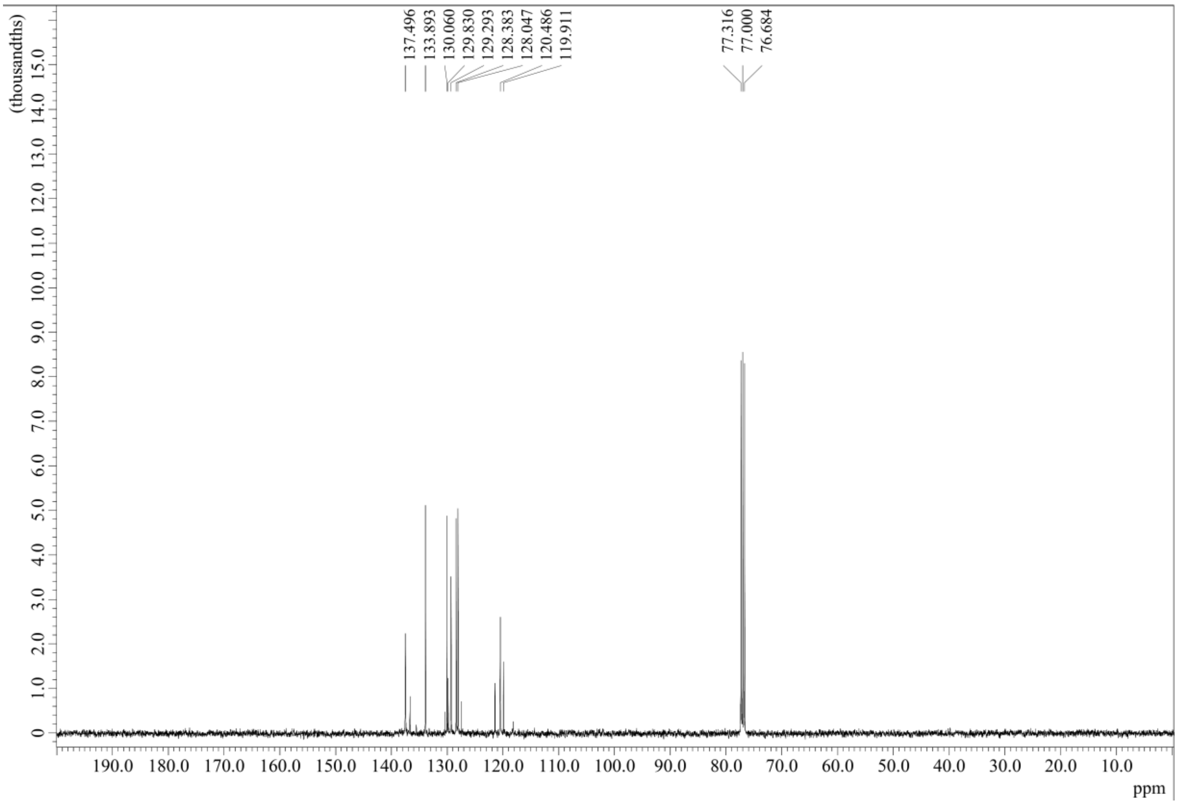

^{1}H NMR of **2a**

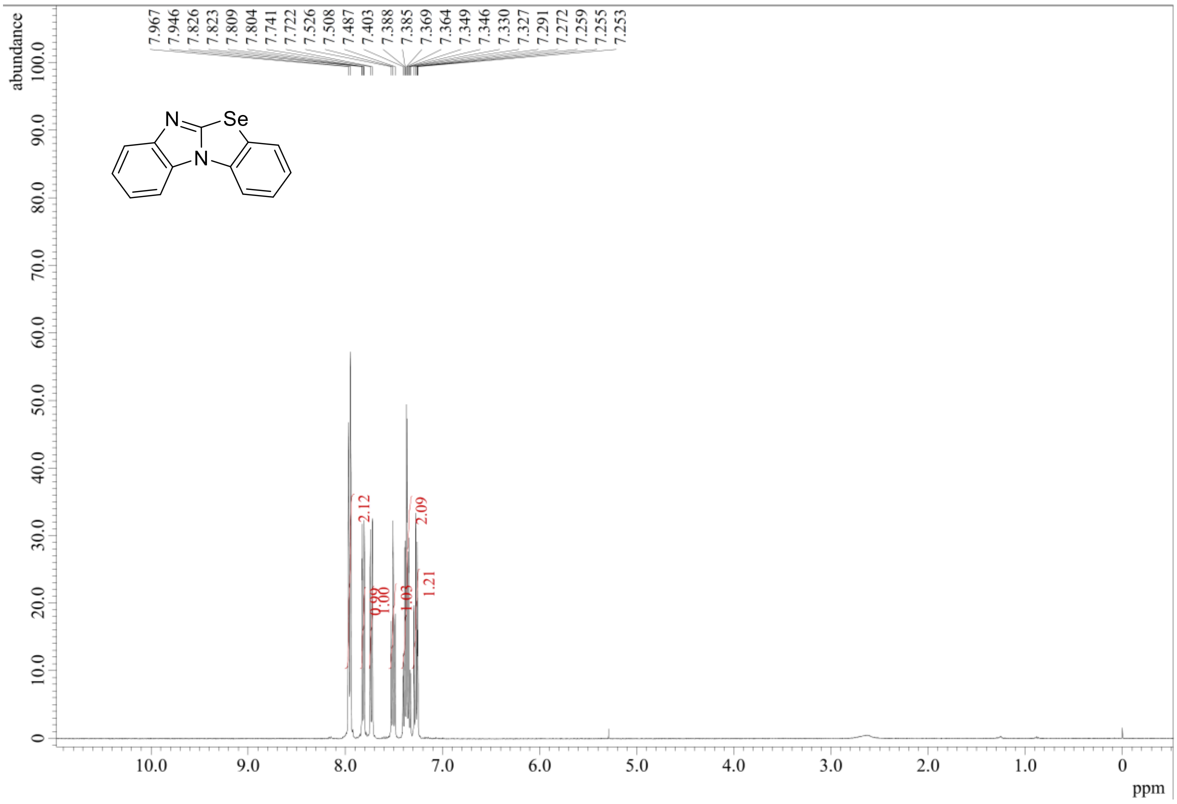

^{13}C NMR of **2a**

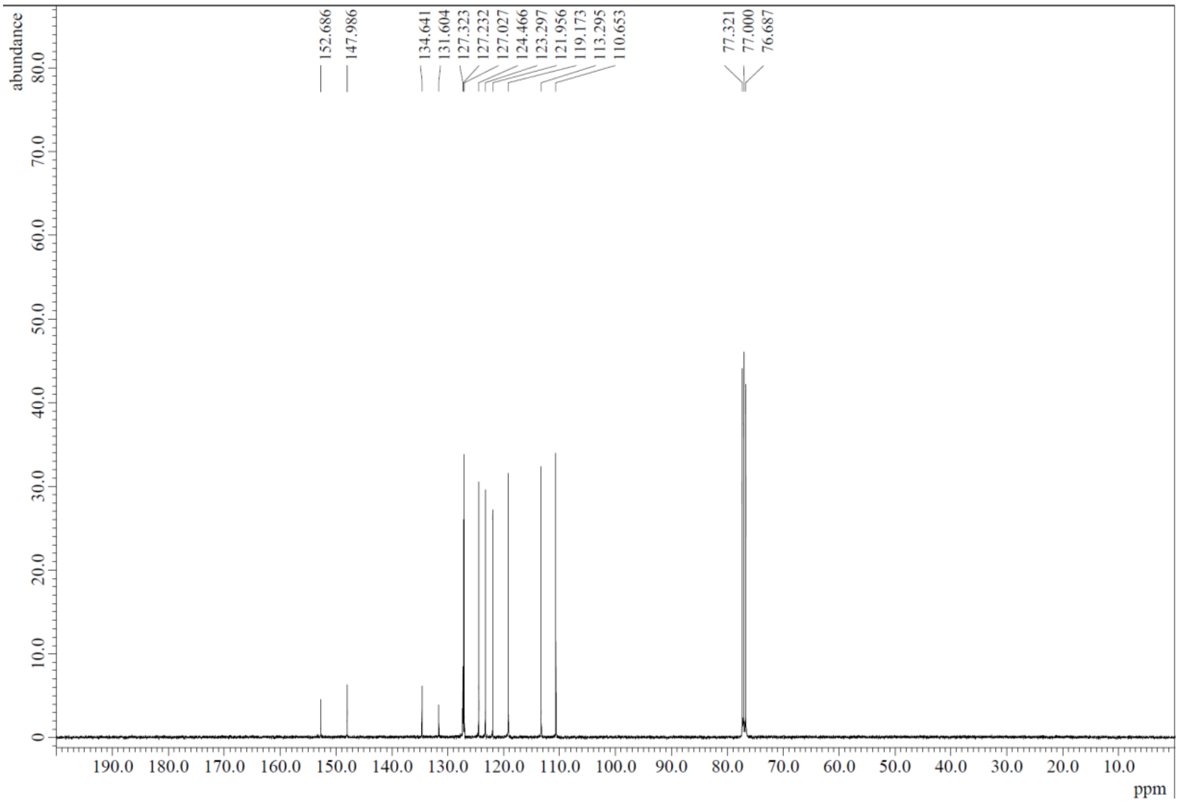

^{1}H NMR of **4**

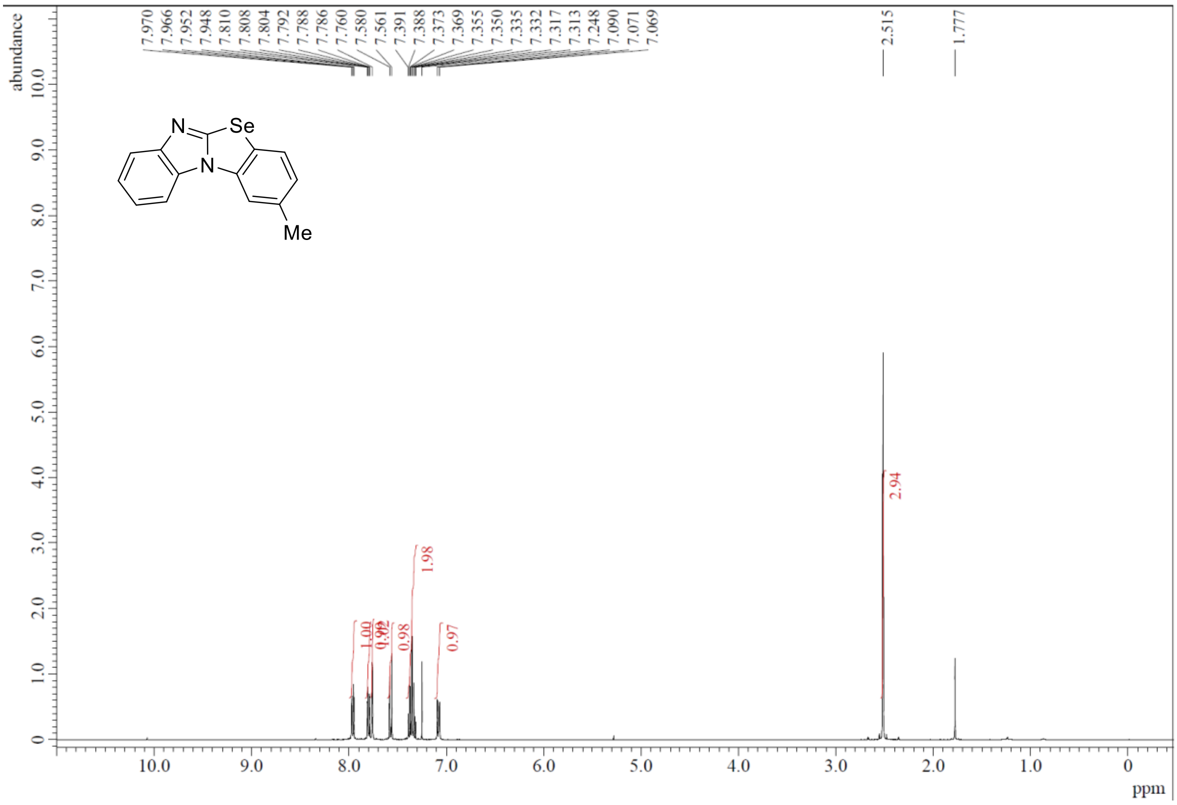

^{13}C NMR of **4**

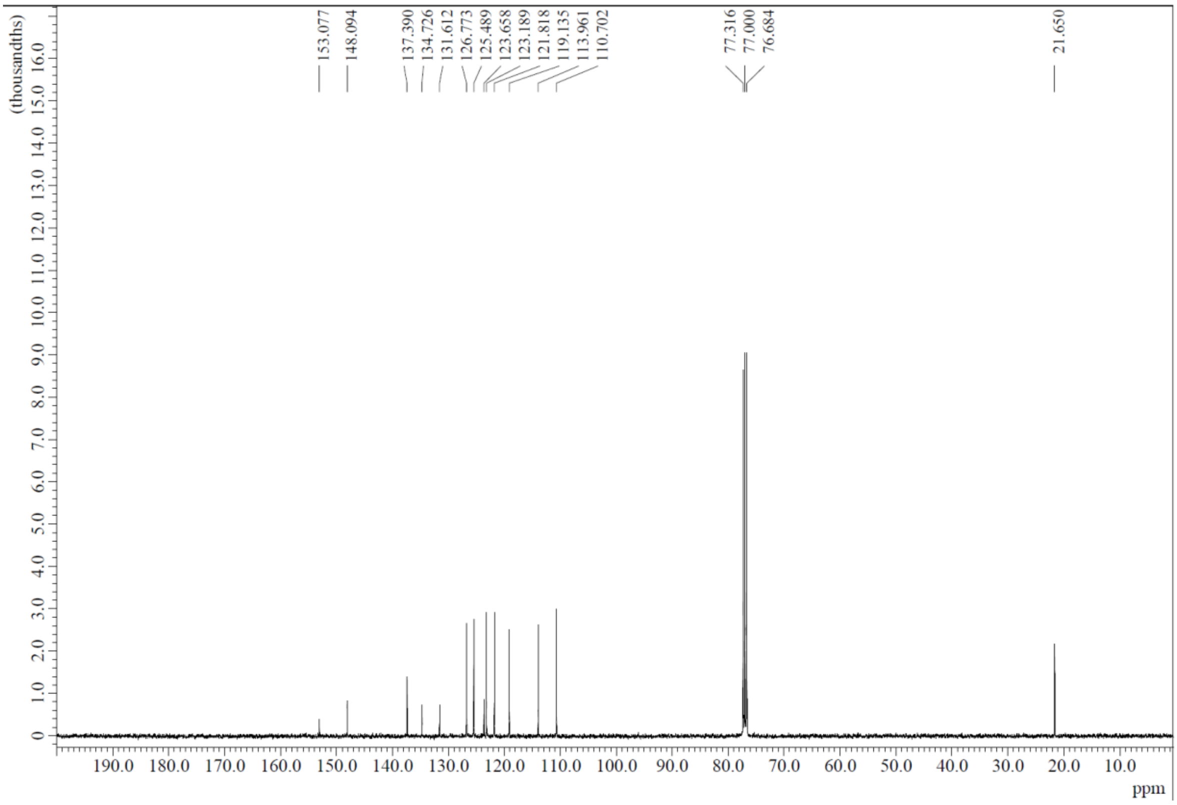

^{1}H NMR of **6**

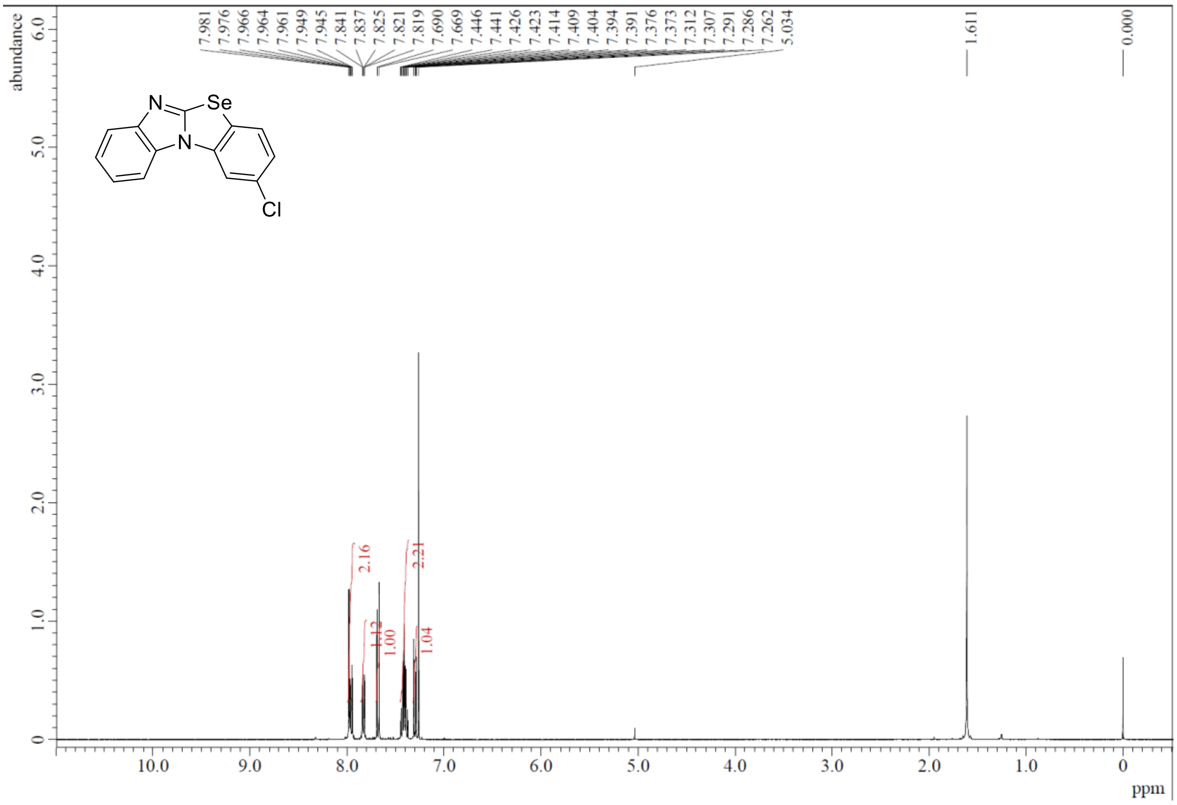

^{13}C NMR of **6**

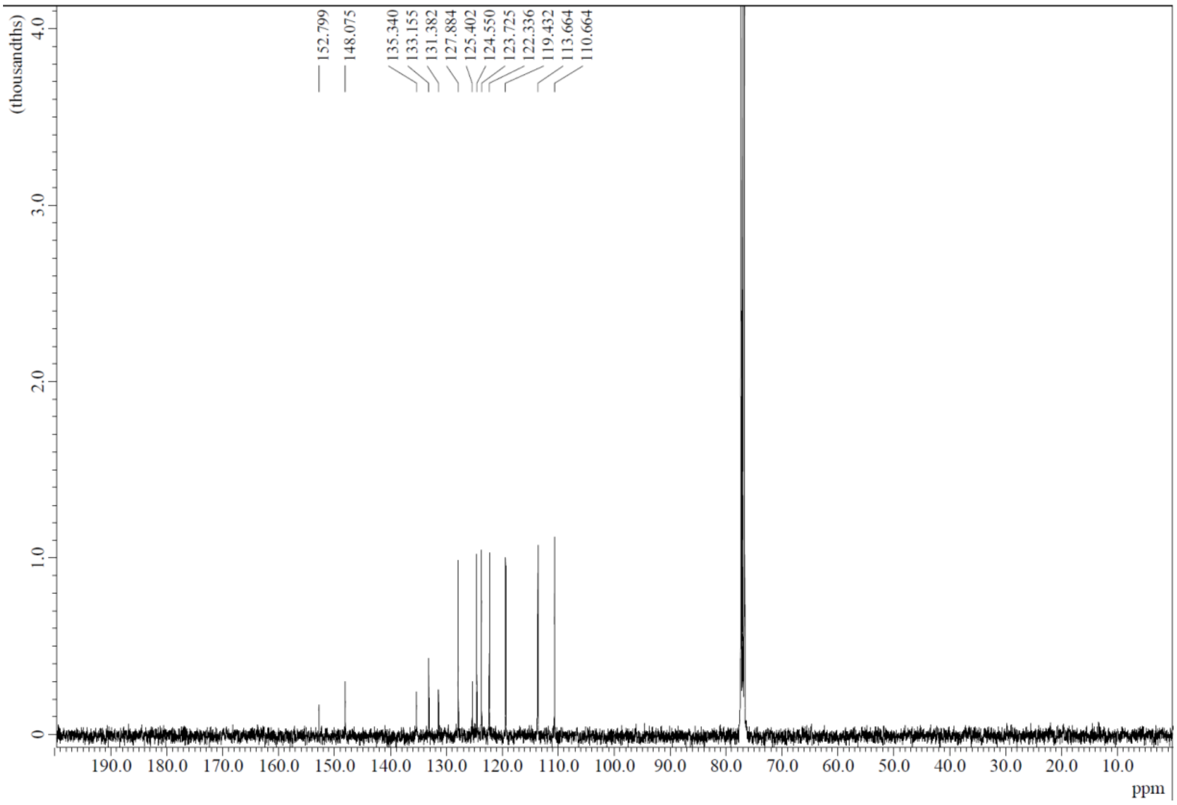

^{1}H NMR of **7**

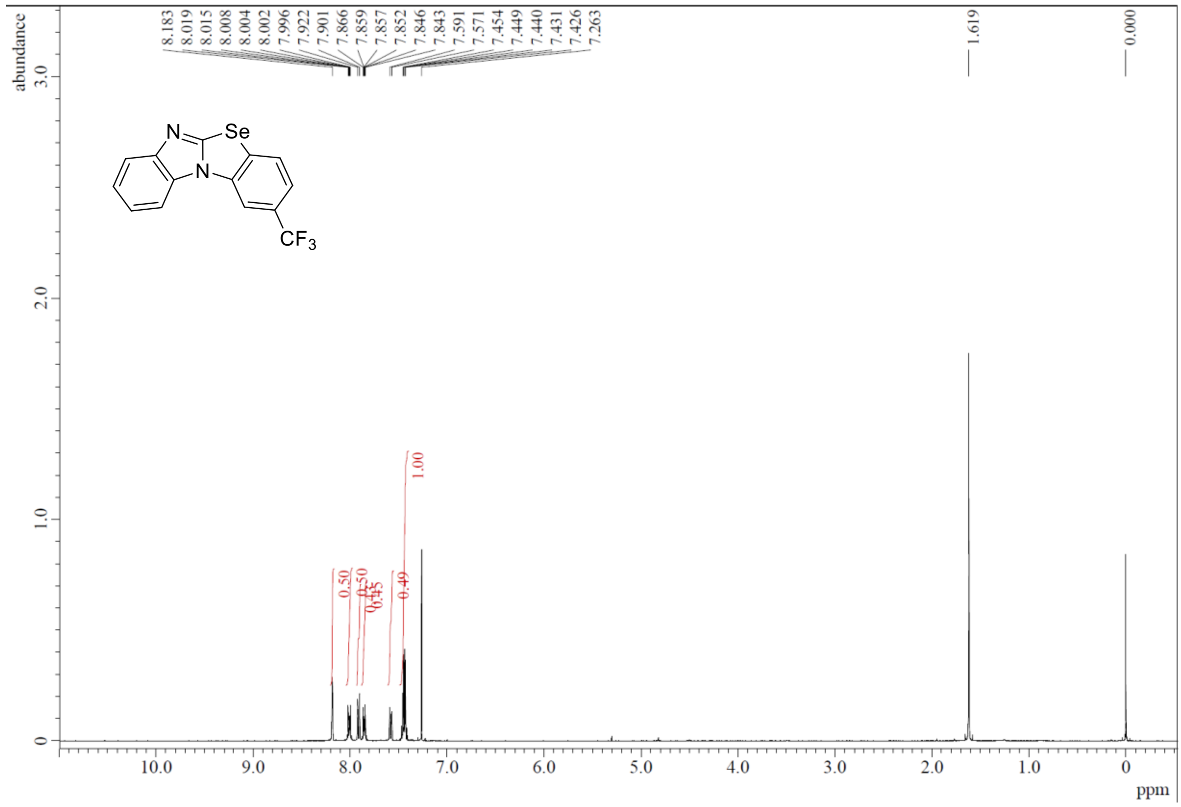

^{13}C NMR of **7**

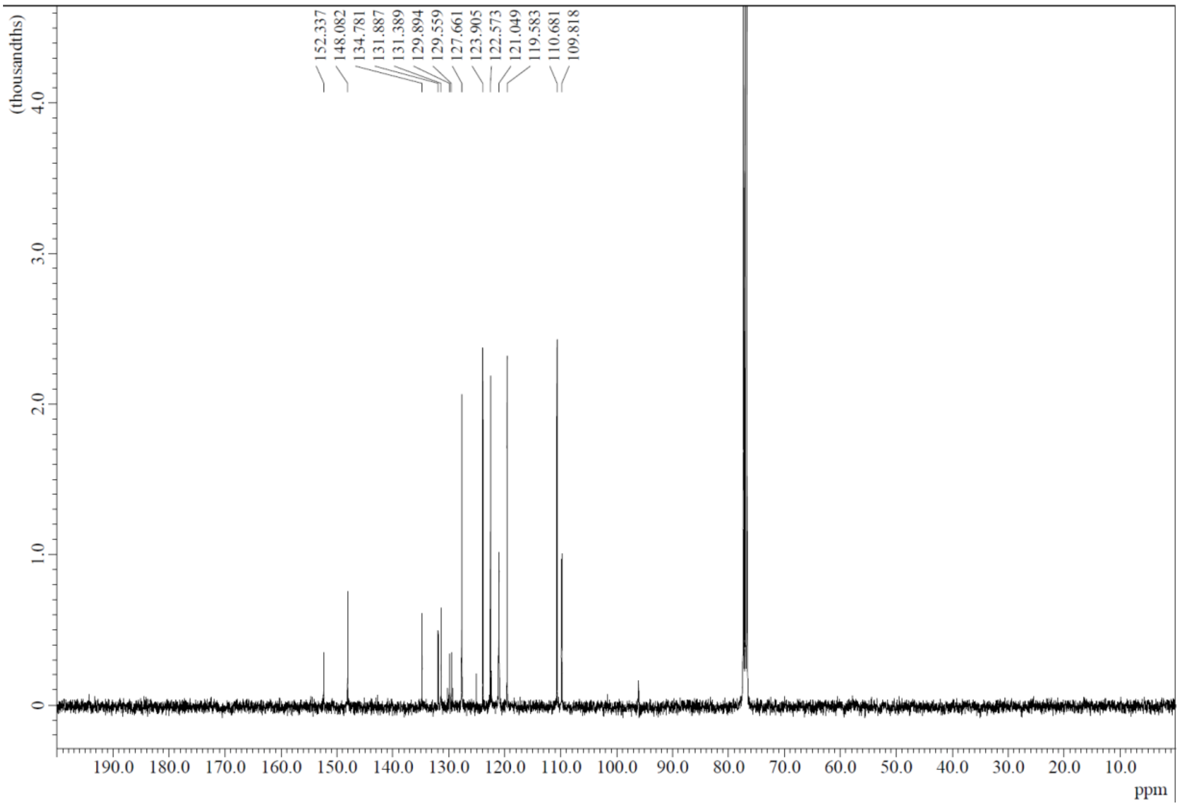

^{1}H NMR of **8**

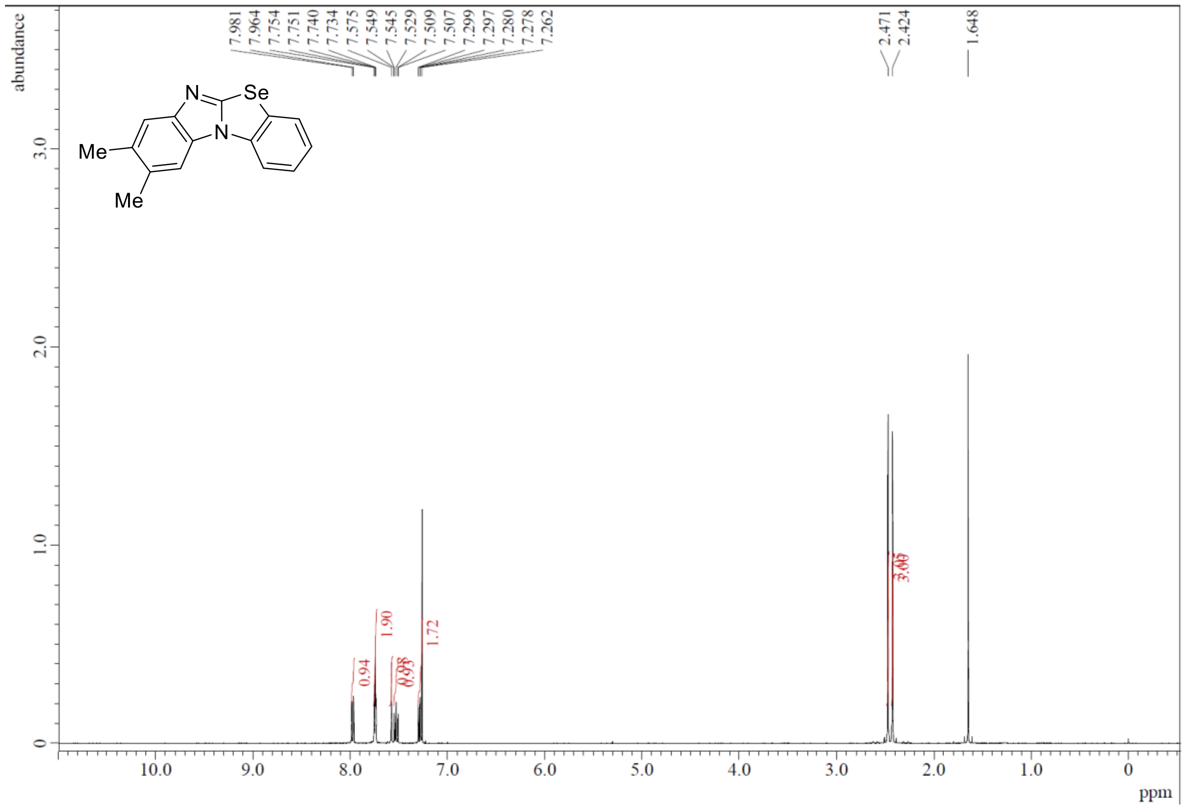

^{13}C NMR of **8**

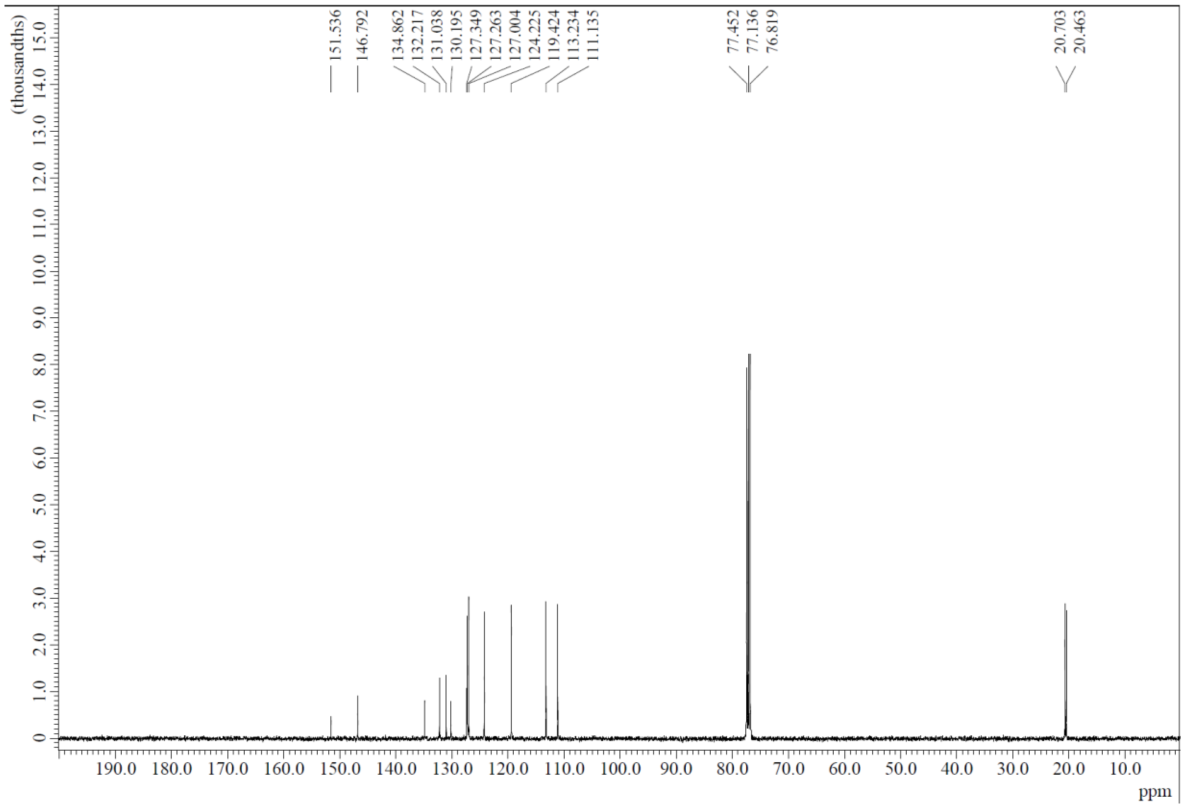

^{1}H NMR of **9**

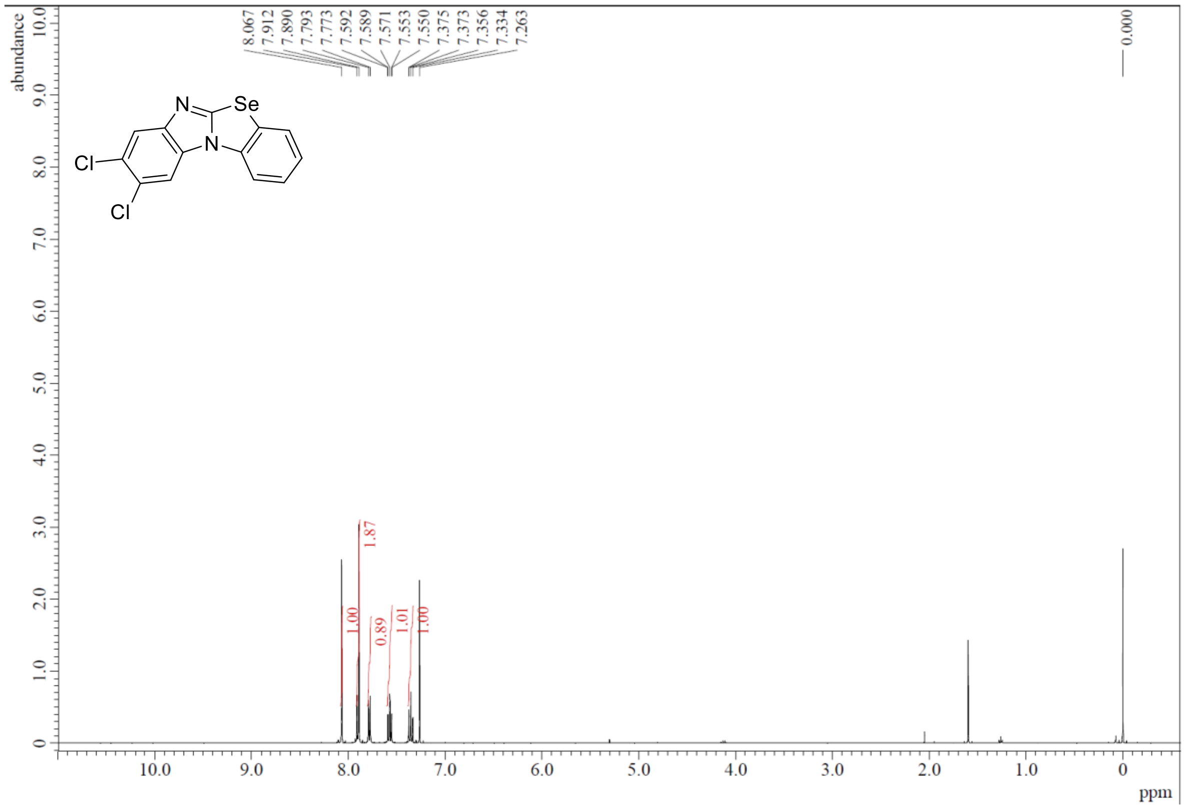

^{13}C NMR of **9**

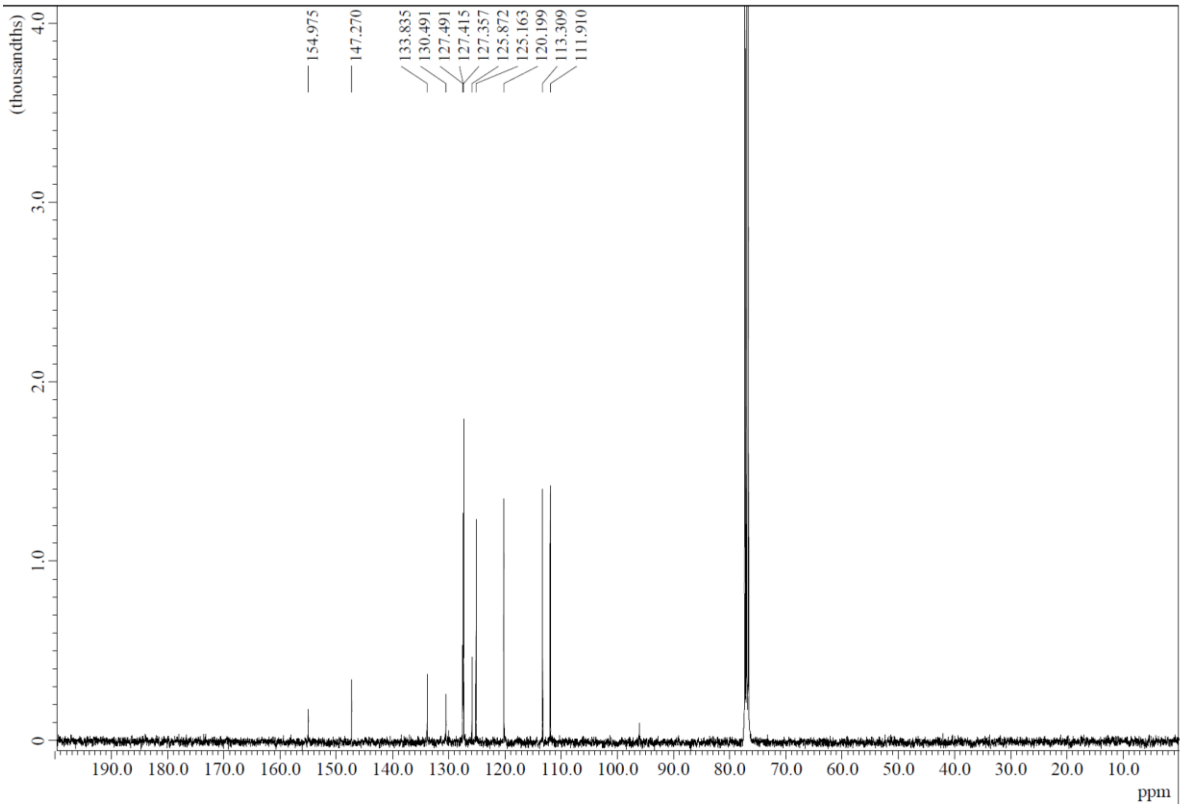

^{1}H NMR of **10**

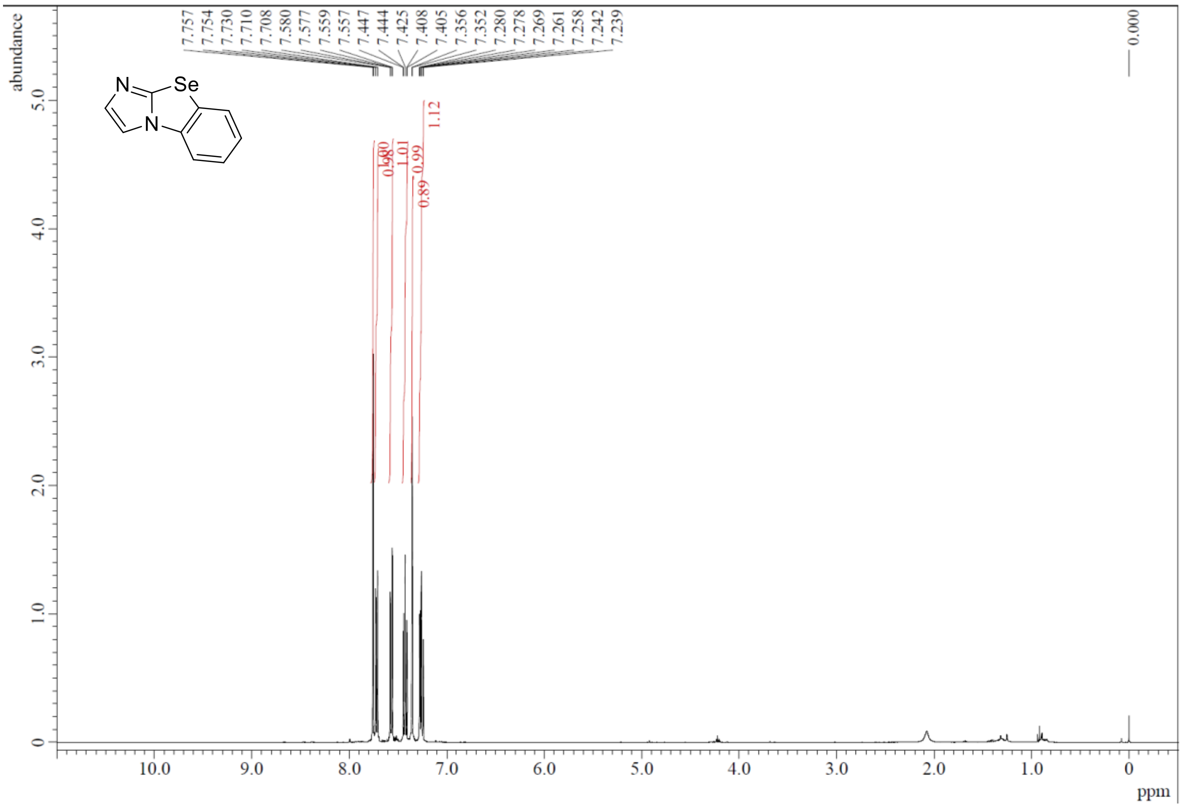

^{13}C NMR of **10**

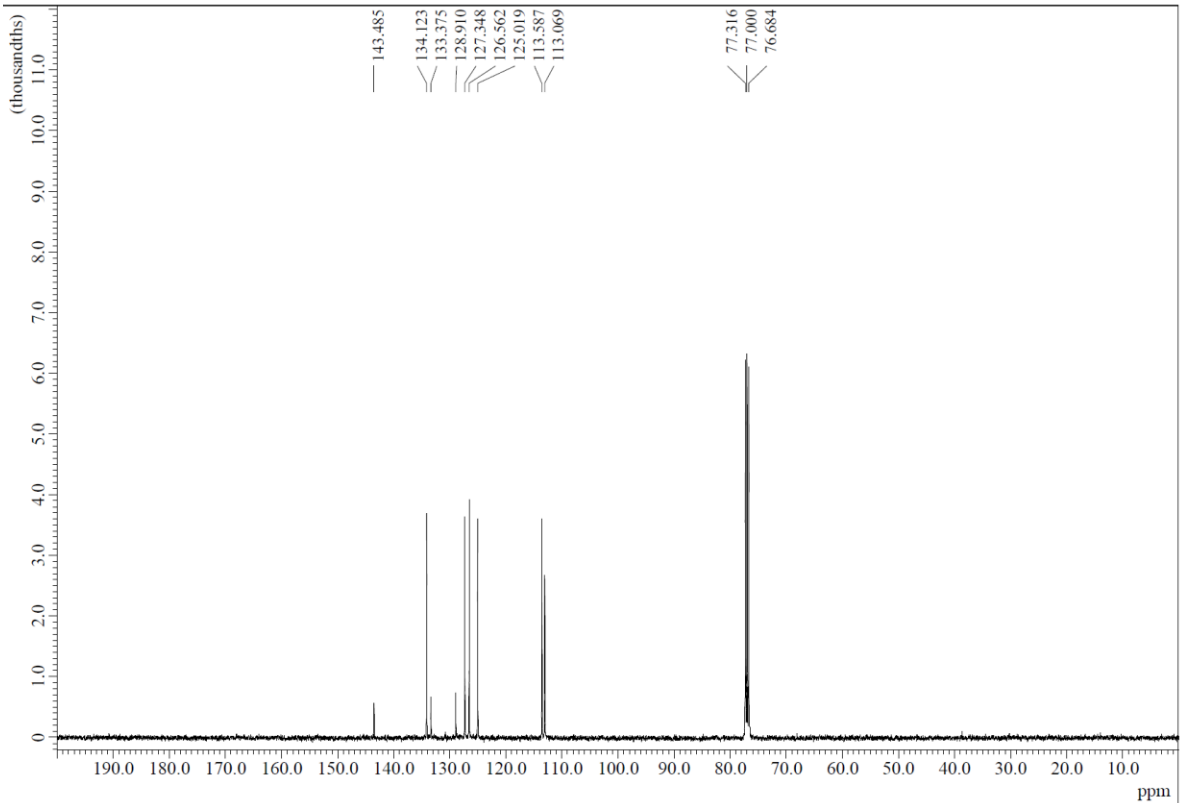

^{1}H NMR of **14**

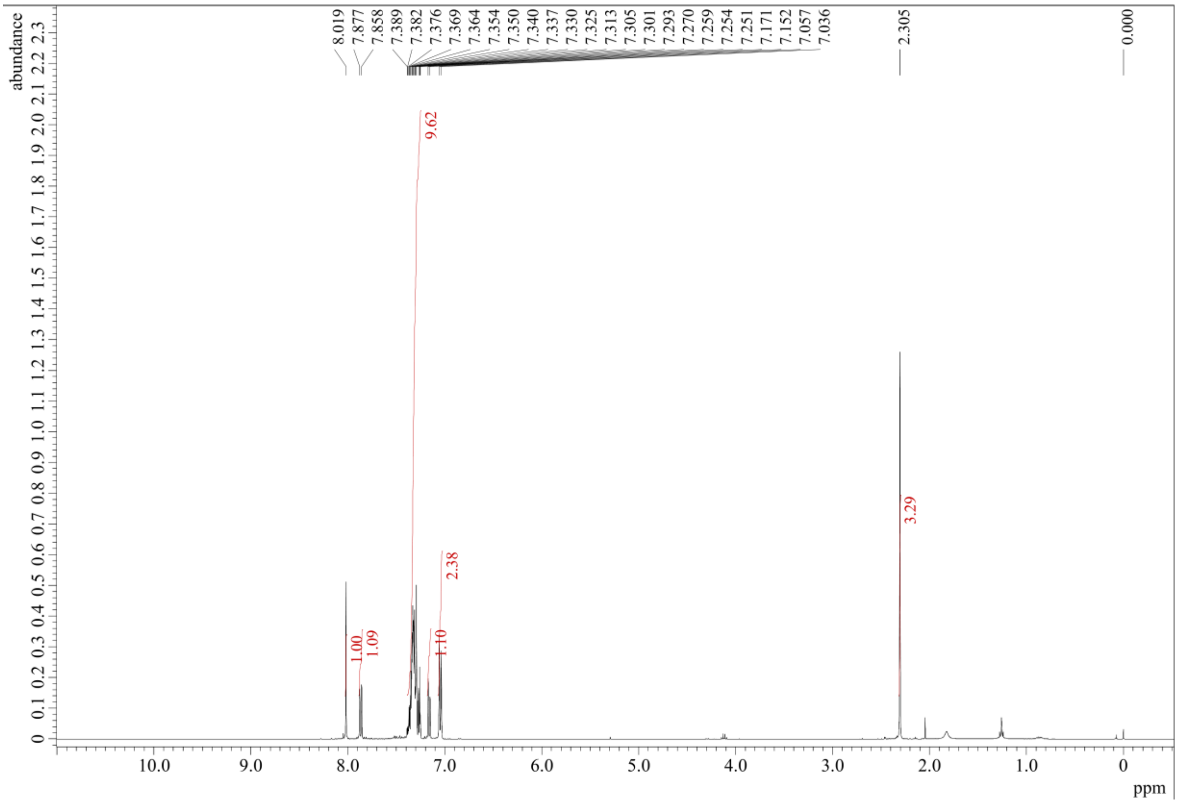

^{13}C NMR of **14**

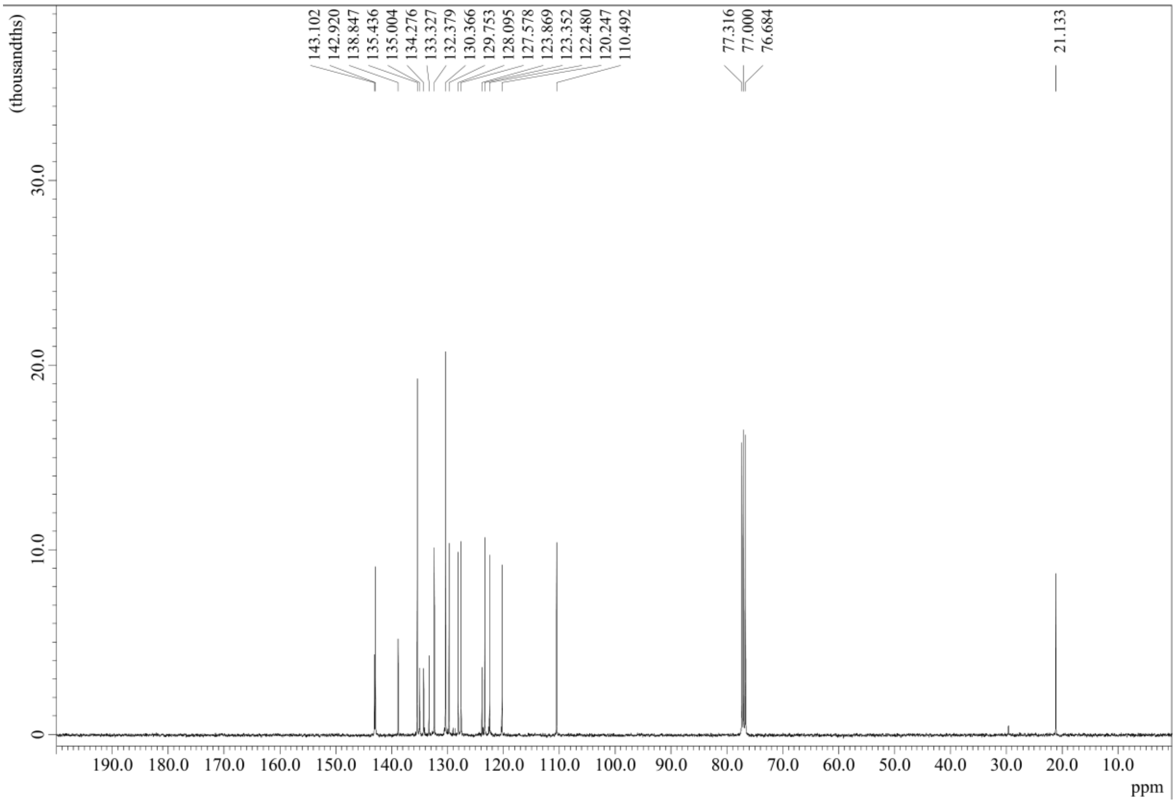

Supplement: File 1 — Experimental details and analytical data, copies of absorption and NMR spectra. [file Beilstein_J_Org_Chem-15-2029-s001.pdf]
